# The genetic architecture of epilepsy across molecular mechanisms and clinical heterogeneity

**DOI:** 10.1002/epi4.70310

**Published:** 2026-08-19

**Authors:** Mohammad Reza Seyedtaghia, Jina Babanzadeh, Marcello Scala, Lorenzo Perilli, Pasquale Striano

**Affiliations:** ^1^ Department of Medical Genetics, Faculty of Medicine Hormozgan University of Medical Sciences Bandar Abbas Iran; ^2^ Tehran Medical Branch Islamic Azad University Tehran Iran; ^3^ Department of Neurosciences, Rehabilitation, Ophthalmology, Genetics, Maternal and Child Health University of Genoa Genoa Italy; ^4^ Medical Genetics Unit IRCCS Istituto Giannina Gaslini Genoa Italy; ^5^ Clinical Pediatrics, Department of Molecular Medicine and Development, Azienda Ospedaliero‐Universitaria Senese University of Siena Siena Italy; ^6^ Department of Neuromuscular Diseases UCL Queen Square Institute of Neurology London UK; ^7^ Pediatric Neurology and Muscular Diseases Unit IRCCS Istituto Giannina Gaslini Genoa Italy

**Keywords:** anti‐seizure medication, drug resistance epilepsy, epilepsy, genomics, seizure

## Abstract

Epilepsy comprises a highly heterogeneous group of neurological disorders unified by a persistent predisposition to recurrent seizures, yet driven by remarkably diverse genetic, molecular, and network‐level mechanisms. Advances in genomic technologies have revealed that epilepsy arises from a multilayered genetic architecture encompassing rare high‐penetrance monogenic variants, common polygenic risk factors, brain‐restricted somatic mosaicism, and extensive gene–environment interactions. These genetic substrates converge on core biological pathways regulating neuronal excitability, synaptic transmission, metabolic homeostasis, neuroinflammation, and circuit development. In this review, we synthesize contemporary insights into the genetic and molecular pathophysiology of seizures and epilepsy, with emphasis on mechanisms that destabilize excitation–inhibition balance, promote epileptogenesis, and drive pharmacoresistance. We highlight how ion channel dysfunction, synaptic vesicle cycling defects, mTOR pathway hyperactivation, glial and metabolic failure, and inflammatory cascades interact to lower seizure threshold and remodel neural networks. Beyond classical monogenic and polygenic models, we discuss the emerging role of somatic mutations, polygenic modifiers of rare variants, and dynamic brain‐state–dependent seizure susceptibility. We further integrate genetic mechanisms with clinical heterogeneity, including age of onset, seizure type, penetrance, pleiotropy, and treatment response, and review translational implications for precision medicine, pharmacogenomics, and emerging molecular therapies such as antisense oligonucleotides, gene regulation strategies, and cell‐based interventions. Understanding the genetic architecture of epilepsy is increasingly informing diagnostic pathways, prognostic stratification, and the development of precision‐based therapeutic approaches.

**Plain Language Summary:**

Epilepsy genetics extends beyond single‐gene disorders and involves multiple interacting layers of genomic variation. In this review, we integrate evidence across rare variants, polygenic risk, somatic mosaicism, and genetic modifiers to provide a broader framework for understanding seizure susceptibility and clinical diversity. By highlighting convergent biological pathways and their effects on neuronal networks, this work supports more refined approaches to epilepsy classification and future precision medicine strategies.

**Key points**

- This review integrates rare, polygenic, and somatic genetic mechanisms into a unified epilepsy framework.
- Multiple genetic architectures converge on shared pathways that destabilize neuronal networks.
- Genetic modifiers help explain penetrance, phenotypic variability, and treatment resistance.
- The review highlights how genomic complexity may refine epilepsy classification.
- Linking genetic architecture to network dysfunction may support future precision therapies.

## 1 INTRODUCTION

Epilepsy stands as one of the most pervasive neurological disorders globally, impacting over 50 million people across varied demographics, with roughly one‐third progressing to pharmacoresistant seizures that resist conventional antiepileptic interventions.^1^ Fundamentally, epilepsy arises from dysregulated neuronal excitability and perturbed neural circuit integrity, culminating in a propensity for recurrent, unprovoked seizures.^2^ Although precipitating factors such as fever, sleep deprivation, or electrolyte imbalances may evoke isolated seizures, true epilepsy signifies an enduring, genetically and neurobiologically anchored elevation in brain excitability.^3^

The elucidation of epilepsy's genetic foundations has accelerated dramatically, propelled by next‐generation sequencing, genome‐wide association studies (GWAS), and expansive cohort analyses.^4^ Heritability metrics from twin research affirm a robust genetic etiology: monozygotic twins exhibit concordance rates far exceeding dizygotic counterparts, implicating genetics in approximately 80% of seizure vulnerability.^5^ Nonetheless, epilepsy's genomic tapestry is profoundly heterogeneous, paralleling its phenotypic pluralism.^6^ Rather than a singular pathology, epilepsy manifests as a spectrum of neurobiological and clinical entities, bound by the cardinal trait of episodic hypersynchrony.

**FIGURE 1:**
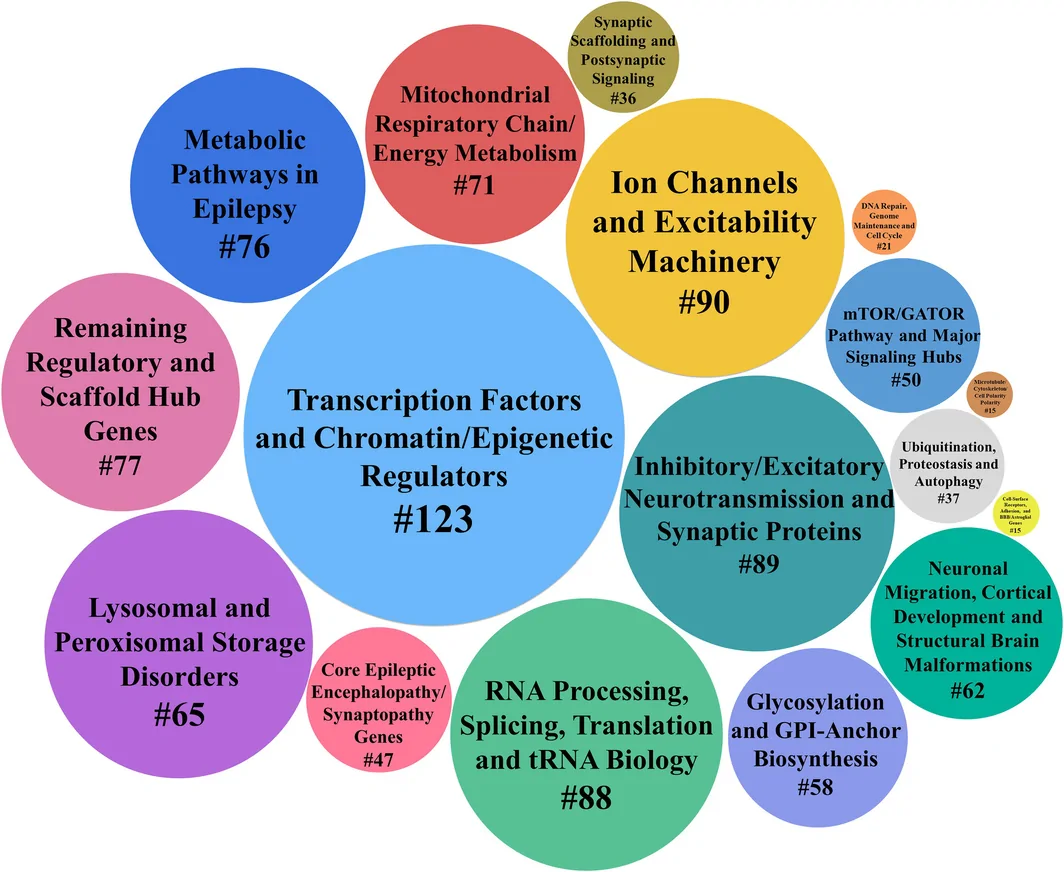
Functional classification of known epilepsy‐associated genes. This figure categorizes 1051 identified epilepsy‐related genes into 18 categories (gene list in Appendix S1). 77 genes are presented in more than one category.

Epilepsy genetics delineates a panoply of inheritance modalities and risk architectures. Presently, exceeding 1000 genes (Figure 1) are validated as monogenic culprits, largely transmitted autosomally dominant.^7^, ^8^ By contrast, prevalent idiopathic generalized epilepsies (IGE) and numerous focal epilepsies conform to polygenic paradigms, wherein susceptibility accrues from legions of common variants—each imparting infinitesimal effects—compounded by background polygenic risk, epigenetic tuning, and environmental modulators.^9^, ^10^ Oligogenic hybrids further complicate the milieu, wherein oligarchies of rare variants synergize with common alleles to dictate onset, trajectory, and refractoriness.^11^

The 2025 International League Against Epilepsy (ILAE) nosology partitions seizures into focal (localized onset), generalized (bilateral symmetric), unknown (ambiguous onset), and unclassified categories, each evincing bespoke genetic signatures.^12^ Heritability diverges strikingly: polygenic contributions (SNP‐based h^2^ up to 40%) prevail in generalized subtypes, whereas focal forms often harbor monogenic rarities or somatic aberrations, underscoring phenotype‐tailored genomic scrutiny.^13^

At the molecular crux, epilepsy genes assault neuronal homeostasis's pillars. Voltage‐gated ion channels dominate, comprising sodium (e.g., *SCN1A/2A* encoding Nav1.1/1.2), potassium (e.g., *KCNQ2/3* for M‐current), and calcium (e.g., *CACNA1A* for P/Q‐type) conduits—responsible for ~40% of monogenic cases. These are shadowed by glutamatergic (e.g., GRIN2A NMDA subunits) and GABAergic (e.g., GABRG2 α2) receptors, synaptic scaffolders (e.g., *STXBP1* for SNARE priming), vesicular handlers (e.g., SV2A), signaling hubs (e.g., *TSC1/2* in *mTORC1* restraint), and transcriptional arbiters (e.g., ARX homeobox).^7^ This convergence illustrates how allelic perturbations unravel the excitation‐inhibition (E/I) dyad, precipitating paroxysmal discharges.

Paradigms evolve beyond heritable germline lesions, implicating somatic mosaicism in focal epileptogenesis. Tissue‐confined mutations, absent peripherally, in scant neuronal fractions can spawn intractable foci.^14^ Deep‐sequencing of malformed cortices discloses that allele burdens as scant as 0.04%–0.07% ignite cascades, exploiting periconceptional circuitry's hypersensitivity to mosaics.^15^

Genotype–phenotype fidelity falters markedly, even intrafamilially.^16^ Incomplete penetrance—sparing subsets of variant bearers—and expressivity gradients—from fleeting benign convulsions to catastrophic encephalopathies—define the variance.^17^ Polygenic risk scores (PRS), epigenetic imprints, and gene‐by‐environment cross‐talk orchestrate this; PRS notably titrates rare variant potency and ferocity, evoking a stratified ontology: rares prime ignition, commons calibrate conflagration.^16^

Translationally, epilepsy genomics heralds a precision era. Genotyping steers pharmacotherapy (e.g., Na^+^ blockers for SCN1A LoF, contraindicated in GoF), anticipates toxicities (e.g., HLA‐B*1502 pre‐screening for aromatic ASMs), and catalyzes bespoke modalities like ASOs, AAV‐gene augmentation, or base editors.^18^, ^19^

This review amalgamates frontier genetic tenets with epilepsy's phenotypic kaleidoscope, probing genomics' alchemy in diagnostics, correlative blueprints, and therapeutic horizons. By unraveling genetic sinews of seizure proneness and defiance, we forge blueprints for prophylactic and reparative stratagems against this encumbering scourge.

## 2 PATHOPHYSIOLOGY OF SEIZURE DISTURBANCE IN NEURAL CIRCUITS

Seizures arise from a self‐reinforcing disruption of neural circuit dynamics, in which multiscale molecular and cellular perturbations converge to destabilize the excitation–inhibition (E/I) balance that maintains brain homeostasis.^3^ This section delineates the multifaceted pathophysiology, encompassing synaptic, ionic, structural, glial, inflammatory, and organellar derangements that converge to lower the seizure threshold and perpetuate epileptogenesis.

### 2.1 Excitation–inhibition collapse and seizure emergence

**FIGURE 2:**
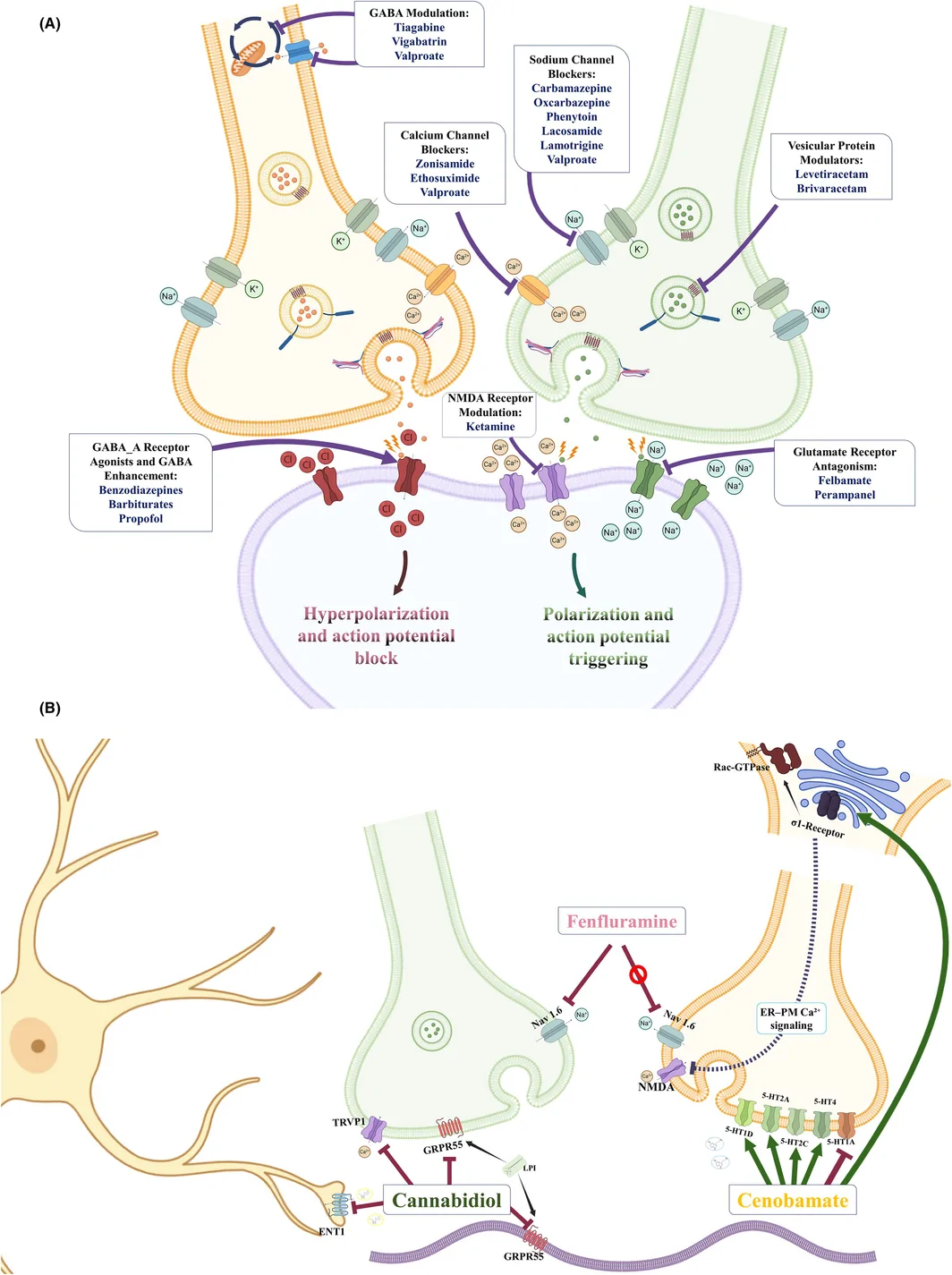
Excitation‐inhibition balance in epilepsy pathophysiology and antiepileptic drugs. (A) This figure depicts the synaptic cleft at the axonal terminal junction between two neurons—one excitatory (green) and one inhibitory (red)—converging on a dendritic terminal (purple). Hyperexcitability of the purple neuron is associated with seizure activity. Excessive glutamatergic excitatory activity coupled with reduced GABAergic inhibitory activity precipitates seizures. Antiepileptic drugs counteract this imbalance by either attenuating excitatory system function or enhancing inhibitory system function. (B) The figure illustrates that cannabidiol primarily antagonizes neuronal membrane GPR55 receptors to block proconvulsant LPI signaling, with secondary desensitization of TRPV1 channels and supporting inhibition of adenosine uptake via ENT1 to enhance inhibitory tone. Fenfluramine acts through a dual primary mechanism: It increases synaptic serotonin by inhibiting SERT, thereby activating ‐5HT2A/2C receptors on GABAergic interneurons to boost inhibition, and it positively modulates sig‐ma1 (σ1) receptors coupled to NMDA receptors to reduce calcium influx and glutamatergic excitation. Cenobamate has a bimodal primary mechanism, selectively inhibiting persistent sodium current (INaP) via Nav1.6 on excitatory neurons while sparing Nav1.1‐dependent interneuron firing, and positively modulating GABAA receptors at a non‐benzodiazepine site to enhance phasic and especially tonic GABAergic inhibition.

Seizures manifest as the clinical and electrographic hallmark of a collapsed E/I equilibrium, enabling unchecked hypersynchronous activity (Figure 2).^3^ In the healthy brain, excitatory glutamatergic pyramidal neurons—expressing ionotropic receptors from *GRIA1*–*GRIA4* (AMPA), *GRIN1*, *GRIN2A*–*GRIN2D* (NMDA), and *GRIK1*–*GRIK5* (kainate) genes—drive synaptic plasticity and information flow. These are counterbalanced by diverse GABAergic interneurons (expressing *PVALB*, *SST*, *VIP*, *NPY*, *CCK*, *CALB1*, and *CALB2*), which enforce spatiotemporal control.^20^ Disruptions via excess excitation, deficient inhibition, or both unleash propagating discharges characteristic of epilepsy.^3^ From a systems perspective, these alterations ultimately converge on network hypersynchrony: the electrophysiologic hallmark of epileptic activity.

### 2.2 Glutamatergic and GABAergic neuron dysregulation

Excitatory dysregulation centers on aberrant glutamatergic transmission. Seizure onset features extracellular glutamate accumulation from heightened release and impaired uptake.^21^ Astrocytic transporters *SLC1A3* (EAAT1/GLAST) and *SLC1A2* (EAAT2/GLT‐1) are profoundly downregulated in human temporal lobe epilepsy and models, while neuronal *SLC1A1* (EAAC1) is upregulated, extending glutamate's synaptic persistence.^21^ Gain‐of‐function mutations in *GRIN1*, *GRIN2A*, *GRIN2B*, and *GRIN2D* prolong NMDA receptor kinetics and Ca^2+^ influx; conversely, loss‐of‐function in *GRM1* and *GRM5* disrupts metabotropic feedback.^22^, ^23^ Ceftriaxone‐mediated *SLC1A2* restoration offers robust neuroprotection in preclinical paradigms.^24^ Two‐photon imaging studies further demonstrate centrifugal glutamate surges from seizure onset zones, accompanied by centripetal collapse of GABAergic restraint, highlighting failure of feed‐forward inhibition.^25^

Inhibitory networks suffer disproportionate attrition.^26^ Recurrent seizures trigger apoptosis in hippocampal *PVALB*‐, *CALB1*‐, and SST‐positive subsets.^27^ Human epileptic single‐nucleus RNA‐seq reveals peak dysregulation in *PVALB*, *SST*, *FEZF2* (layer 5–6 pyramidal), and *CUX2* (layer 2–3 pyramidal) clusters, including *SHISA9* upregulation that amplifies *GRIA1*–*GRIA4* currents.^28^ Mutations in GABA_A subunits (*GABRA1*, *GABRB3*, *GABRG2*, *GABRD*, *GABRQ*) compromise trafficking, stability, or Cl^−^ flux, underpinning GEFS+ and juvenile myoclonic epilepsy.^28^ Collectively, these findings indicate that the selective vulnerability of inhibitory interneuron networks represents a central driver of circuit‐level hyperexcitability in both genetic and acquired epilepsies.

### 2.3 Ion channelopathies in epilepsy

Ion channel disorders dominate monogenic epilepsies.^29^ Voltage‐gated Na^+^ channels (*SCN1A*/Nav1.1, *SCN2A*/Nav1.2, *SCN8A*/Nav1.6; β‐subunit *SCN1B*) yield gain‐ or loss‐of‐function; *SCN1A* haploinsufficiency in Dravet syndrome selectively impairs *PVALB*+ interneurons.^30^ K^+^ channels like *KCNQ2/3* disrupt M‐currents in neonatal seizures, *KCNT1* hyperactivates in migrating focal seizures, and *KCNA1/2* variants cause encephalopathies.^31^ Ca^2+^ channels (*CACNA1A*/Cav2.1, *CACNA1G*/H T‐type, *CACNB4*) drive absence and ataxic syndromes, while *CLCN2* Cl^−^ channel mutations shift reversal potentials in idiopathic generalized epilepsy.^32^, ^33^ Importantly, the phenotypic diversity associated with channelopathies reflects cell‐type–specific expression patterns and divergent biophysical consequences of gain‐ versus loss‐of‐function variants.

### 2.4 Synaptic vesicle cycling and neurotransmitter release machinery

Vesicle dynamics are pivotal for transmitter homeostasis. VGLUTs (*SLC17A6*–*SLC17A8*) and VGAT (*SLC32A1*) partner with SV2A; SV2A variants precipitate intractable encephalopathies, often refractory to levetiracetam.^34^, ^35^ SNARE components (*STX1B*, *STXBP1*, *SNAP25*) and DNM1 mutations derail priming and release in infantile epilepsies.^36^

### 2.5 mTOR pathway activation and structural circuit remodeling

Chronic hyperexcitability entrenches via structural plasticity. Mossy fiber sprouting in the dentate gyrus, fueled by mTORC1 hyperactivation from *TSC1/2*, *DEPDC5*, *NPRL2/3* loss, aberrantly rewires circuits.^37^ Acute *Tsc1* knockout in adults evokes rapid seizures, highlighting mTOR's epileptogenic force.^38^ Effectors like 4E‐BP1/2 and *RPS6KB1* (S6K) boost translation, phosphorylating FMR1 to escalate excitability proteins.^39^ At the network‐level, mTOR‐driven structural remodeling promotes the formation of recurrent excitatory loops that lower seizure threshold and facilitate chronic epileptogenesis.

### 2.6 Glial network disruption and metabolic dysfunction

Glia and metabolism amplify circuit vulnerability. Astrocytic GJA1 (Cx43) and PANX1 undergo seizure‐evoked phosphorylation, disrupting coupling and unleashing hemichannels.^40^ Mitochondrial insults from *POLG*, *TWNK*, *SUCLA2*, or mtDNA variants (e.g., *MT‐TL1* m.3243A>G in MELAS; *MT‐TK* m.8344A>G in MERRF) impair respiration, fostering ROS and damage.^41^

### 2.7 Neuroinflammatory cascades and blood–brain barrier breakdown

Inflammation now ranks as a primary epileptogenic driver. Seizures mobilize microglia/astrocytes, elevating NLRP3, IL1B, and TNFA; IL‐1β heightens excitability through *GRIN2B* phosphorylation.^42^ Kynurenine pathway induction (IDO1, KMO) yields excitotoxic quinolinic acid. BBB compromise via *CLDN5*, *OCLN*, and *AQP4* dysregulation enables albumin leakage and *TGFB1*‐induced gliosis.^42^ Epigenetic shifts, including *MIR134*‐mediated *LIMK1* silencing and ubiquitin defects (*UBE3A*, *HUWE1*, *FBXW7*), entrench proteostasis failure, alongside CAMK2A/B autophosphorylation post‐status.^43^, ^44^, ^45^

### 2.8 Other mechanisms

Supplementary pathways erode seizure thresholds independently of core E/I imbalance. DNA repair deficits (*OGG1*, *APEX1*, *MUTYH*, *POLG*) accrue mtDNA oxidative damage, eroding bioenergetics and igniting inflammasomes.^46^ Chaperone collapse (HSPA1A/8, HSP90AA1; DNAJB6, BAG3) fosters aggregation of channels (*SCN1A*, *KCNQ2*) and receptors (*GABRA1*, *GRIN2B*), yielding toxic dysfunction.^47^ Ciliary anomalies (*AHI1*, *CEP290*, *TMEM67/216*, *IFT88*, *BBS1–12*) derail SHH/PTCH1/SMO, Wnt, mTOR, and interneuron/adenosine signaling, engendering hyperexcitable foci in ciliopathies like Joubert syndrome.^48^

In aggregate, epilepsy emerges from the synergistic convergence of genetic, synaptic, metabolic, inflammatory, structural, and organellar disturbances that collectively destabilize neural networks and prime the brain for hypersynchronous activity. Table S1 shows major epilepsy‐associated genes by functional category.

## 3 SEIZURE TRIGGERS AND THE ROLE OF GENETIC VARIATION IN SEIZURE OCCURRENCE

Seizure occurrence reflects dynamic interactions between intrinsic genetic vulnerability and extrinsic precipitants that collectively lower the threshold for hypersynchronous activity.

### 3.1 The concept of seizure threshold and individual susceptibility

**FIGURE 3:**
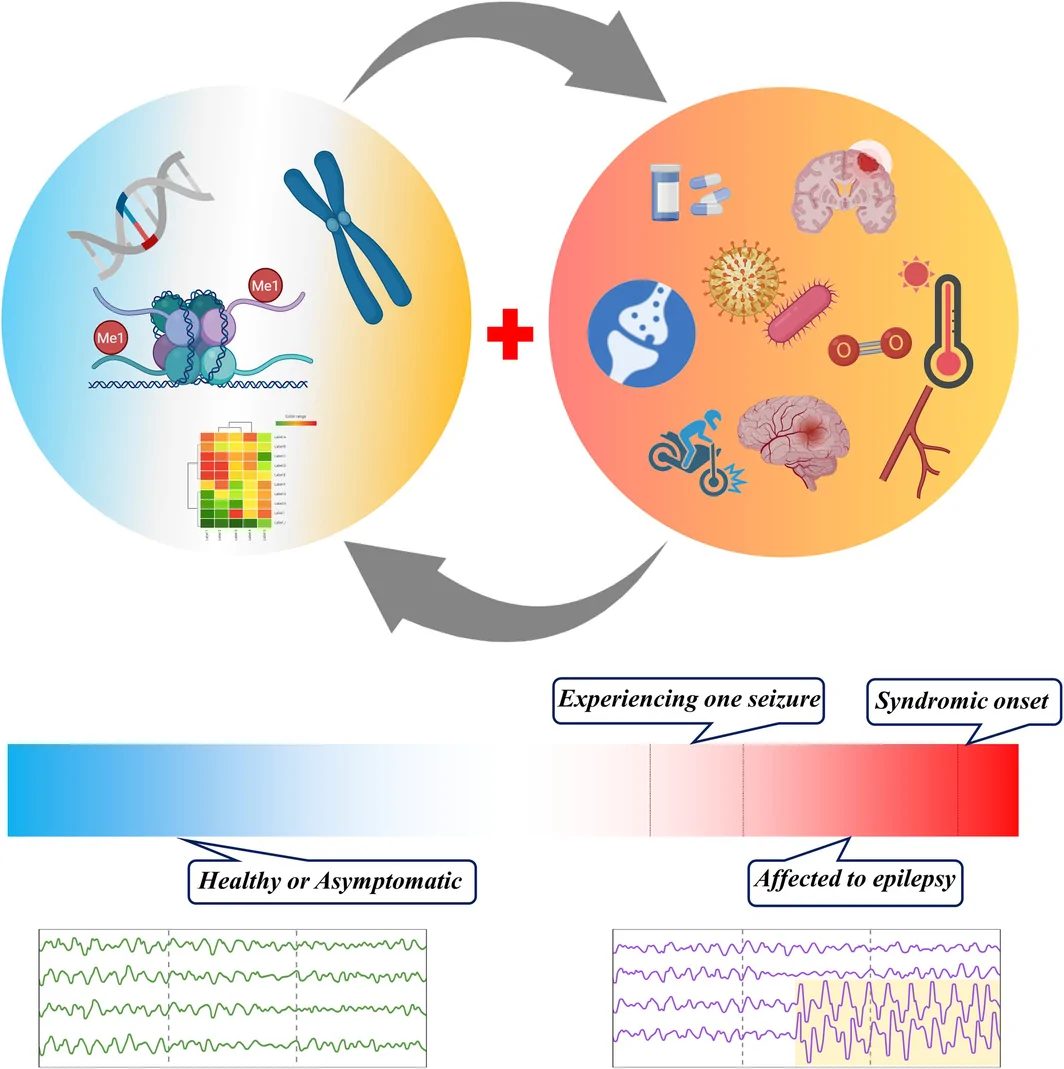
Gene–environment interactions in seizure and epilepsy pathogenesis. Both genetic and environmental factors contribute to an individual's susceptibility to seizures and epilepsy. Some individuals may possess genetic or environmental profiles that preclude any seizure events. Others with specific genetic backgrounds may resist seizures despite environmental triggers. However, genetically predisposed individuals may experience their first seizure upon encountering an environmental precipitant. In more severe cases—particularly those involving comorbidities beyond epilepsy—genetic factors predominate, where an inherited variant can produce profound symptoms independent of environmental triggers. Beyond direct physiological effects, environmental factors may induce epigenetic modifications that alter gene expression.

Seizures represent episodes of excessive neuronal synchronization and affect approximately 8% of the population over a lifetime.^1^ The “seizure threshold”—a measure of individual propensity—exhibits substantial inter‐ and intra‐individual variability. Some individuals experience spontaneous unprovoked seizures in the absence of external triggers, whereas others remain resistant even to potent precipitants, underscoring a strong genetic contribution (Figure 3).^1^, ^3^, ^49^

This threshold fluctuates temporally, influenced by brain states like sleep–wake cycles, hormonal shifts (estrogen/progesterone), circadian rhythms, metabolism, and seizure history.^50^ Genetic variants shape both baseline resilience and sensitivity to these modulations, dictating heterogeneous responses.^49^

### 3.2 Environmental and metabolic seizure triggers

In susceptible individuals, diverse insults can reliably provoke seizures, including: hypoxia, hypoglycemia, hyponatremia, trauma, tumors, strokes, vitamin deficits (thiamine/pyridoxine), CNS infections (meningitis/encephalitis), inflammation, autoimmune encephalitis, alcohol withdrawal, eclampsia, and toxins.^2^ Notably, these stressors do not affect most individuals but markedly increase risk in genetically predisposed populations, highlighting gene–environment interplay.

### 3.3 Genetic modulation of trigger responsiveness: specific examples

Genetic variants modulate trigger sensitivity, illustrating how molecular perturbations amplify the impact of environmental stressors.

Hypoxia elicits seizures via gene–environment synergy. The hERG potassium channel enhances neuronal resilience to stressors such as hypoxia and hyperthermia; mutations or downregulation compromise this protective effect.^51^ Hypoxia‐inducible factor (HIF) pathways adapt to deprivation, with variants potentially attenuating compensatory responses.

Febrile seizures—the commonest provoked childhood variant (2%–5% incidence)—arise from abrupt hyperthermia.^52^ *SCN1A* mutations drive susceptibility to febrile seizures and GEFS+.^53^ Mechanistically, Na^+^ channel dysfunction preferentially impairs fast‐spiking GABAergic interneurons, whose high metabolic demands are insufficiently met during hyperthermia, leading to temperature‐dependent loss‐of inhibition.^53^, ^54^

Infections and inflammation incite seizures via pathogen damage, cytokine surges (TNF‐α, IL‐1β, IL‐6) breaching the BBB, excitability enhancement, GABA impairment, and K^+^ efflux.^55^ Polymorphisms in immunity/inflammation loci (IL‐1β, TNF‐α, TLRs) correlate with febrile seizure risk in select cohorts, though reproducibility varies.^53^, ^55^

Metabolic derangements—hyperammonemia, mitochondrial failure, organic acidemias—precipitate seizures through toxicity, energy deficits, and dysregulation.^56^ Ammonia disrupts GABA transmission; *SLC25A15* variants in hyperornithinemia‐hyperammonemia‐homocitrullinuria syndrome heighten vulnerability.^57^ Mitochondrial disorders (mtDNA/nuclear mutations) manifest seizures via ATP scarcity, ROS, and Ca^2+^ imbalance, blending baseline deficits with stress intolerance.^56^

### 3.4 The “Trigger × Gene × Brain State” framework: integration of genetic variation with environmental context

Seizure precipitation is best conceptualized through a tripartite framework comprising: genetic excitability substrate, environmental/metabolic trigger, and brain state (sleep phase, history, hormones, drugs).^58^ This “Trigger × Gene × Brain State” paradigm elucidates phenotypic variability, trigger penetrance, and temporal clustering. For example, *KCNQ2* heterozygotes (loss‐of‐function via dominant‐negative heterotetramers) may develop nocturnal seizures during sleep‐synchronized activity, further exacerbated by fever or sleep deprivation.^58^

### 3.5 Neuronal plasticity and the evolution from “One and Done” to chronic epilepsy

Provoked seizures (acute stressor‐driven) contrast unprovoked ones, with the shift to chronic epilepsy via epileptogenesis—network rewiring and neuronal remodeling that erodes thresholds.^59^ A lone provoked event (hypoxia/trauma/infection) may resolve sans sequelae in resilient individuals or ignite synaptic/neuronal cascades toward recurrence in vulnerable ones.^60^ Pathogenic variants in ion/synaptic genes hasten this progression, while resilience factors (repair, inflammation resolution, homeostasis) avert chronicity despite insults.

## 4 TREATMENT REFRACTORINESS AND ANTI‐SEIZURE DRUG MECHANISMS

Despite major advances in anti‐seizure medication (ASM) development, approximately one‐third of individuals with epilepsy remain pharmacoresistant after adequate treatment trials, highlighting the need for mechanism‐informed therapeutic strategies.^1^ Drug response is influenced by pharmacokinetic and pharmacodynamic factors, including variability in drug metabolism, blood–brain barrier transport, target adaptation, and genetic determinants of efficacy and adverse effects. Integrating molecular pharmacology with genetic and clinical stratification may improve treatment selection and support precision medicine approaches in epilepsy. A schematic overview of ASM targets is presented in Figure 2, and detailed discussion of pharmacokinetic and pharmacodynamic mechanisms is provided in Text S1.

## 5 GENETIC ASPECTS OF EPILEPSY VARIATION

The genetic architecture of epilepsy is highly heterogeneous and is shaped by developmental timing, modes of inheritance, mutation types, and polygenic influences that collectively influence age at onset, severity, and syndromic presentation.

### 5.1 Age of onset: neonatal, infantile, and childhood epilepsies

Seizure onset age often correlates with the underlying etiologic genes, mirroring stage‐specific expression, circuit maturation, and myelination that calibrate excitability.^3^

#### 5.1.1 Neonatal epilepsy (onset ≤28 days of age)

Neonatal seizures afflict 1–3/1000 births, with genetic causes in 35%–45% post‐exclusion of hypoxia, hemorrhage, or infection.^61^ Pivotal genes encompass *KCNQ2/3* (M‐current K^+^ channels), *SCN2A* (Nav1.2 Na^+^ channel), and CDKL5 (synaptic regulator). Benign familial neonatal epilepsy (BFNE) stems mainly from *KCNQ2/3* mutations, debuting <5 days. In *KCNQ2*, truncations evoke haploinsufficiency for self‐limited BFNE, while pore/voltage‐sensor missense variants precipitate severe DEEs.^61^ Table S3 enumerates high‐penetrance DEE genes, inheritance, onset, and phenotypes.

#### 5.1.2 Infantile epilepsy (onset 1–12 months of age)

Infancy's explosive myelination/synaptogenesis harbors BFIE (*PRRT2* mutations, 3–20 months onset; presynaptic release modulator; autosomal dominant, benign).^62^ Infantile spasms (West syndrome; 4–8 months) yield genetic hits in 20%–30%, notably *TSC1/2*.^63^ *CDKL5* (early DEEs ~1–2 weeks) and X‐linked *ARX* (spasms with disability) also feature.^63^

#### 5.1.3 Childhood epilepsy (onset 1–15 years)

Childhood epilepsy peaks with diverse genetics. Polygenic CAE emerges 4–8 years.^64^ Dravet syndrome, infancy‐onset but childhood‐persistent, shows fever/photosensitivity/resistance; de novo *SCN1A* mutations dominate 80%–90%.^64^

### 5.2 Penetrance and pleiotropy

#### 5.2.1 Incomplete penetrance in monogenic epilepsies

Incomplete penetrance pervades monogenic epilepsies; GEFS+ twin/family data peg ~60%, sparing 40% carriers.^65^ Polygenic risk scores (PRS) modulate: higher PRS carriers manifest seizures/severity atop identical variants.^66^ Thus, monogenic forms interweave polygenic/gene–environment factors, refining counseling/risk models.

#### 5.2.2 Pleiotropy: phenotypic spectrum within single genes

*SCN1A* exemplifies pleiotropy, spanning GEFS+ to Dravet.^67^ Loss‐of‐function (nonsense/frameshift) drives severity; gain/dominant‐negative milder.^68^ Biophysics—channel impairment, localization, cell specificity—correlate variability. Exon/transcript effects amplify: neonatal exon 5 N mutations hasten/severe DEEs; adult exon 5A milder/later, spotlighting isoform‐targeted interventions.

### 5.3 Inheritance patterns: familial and de novo variants

#### 5.3.1 De novo mutations and developmental and epileptic encephalopathies

De novo variants fuel ~86% DEEs via gametogenesis/embryogenesis, curtailing fitness/familiality.^69^ *STXBP1*, *CDKL5*, *SCN8A* show elevated de novo rates.

#### 5.3.2 Inherited pathogenic variants and variable inheritance

Autosomal dominant with incomplete penetrance/variable expressivity propagates milder monogenic epilepsies; compound heterozygous/homozygous intensify. Recessive rarer in metabolic forms (e.g., *SLC25A22* mitochondrial glutamate transport). X‐linked (*CDKL5*, *ARX*) skew female phenotypes via inactivation; *PCDH19* heterozygotes alone affected, severity X‐inactivation modulated.^8^, ^70^

#### 5.3.3 Gonadal mosaicism

Gonadal mosaicism—germline‐restricted variants undetected somatically—underpins >3% “de novo” cases, per sibling recurrences/deep sequencing; vital for counseling unaffected parents.^71^

### 5.4 Seizure types: focal versus generalized; secondary hit model

Genetic architectures diverge: Rare monogenic variants (particularly in ion channel and synaptic genes such as *SCN1A* and *KCNQ2*) are relatively uncommon in the more prevalent epilepsy syndromes; polygenic via GWAS h^2^_SNP (Epi25/ILAE 2023–2025) interplay common variants/environment.^12^, ^13^ Focal: <5% monogenic (e.g., *CHRNA4*/*CHRNB2* nocturnal frontal), h^2^_SNP 9%–16%, PRS familial enrichment, neurodev correlations. Generalized: 1%–2% monogenic (GABRG2 GGE), h^2^_SNP 30%–40%, familial high, top‐decile PRS OR 4.6. Unknown: <3% monogenic, h^2^_SNP 15%–25%; unclassified minimal (<1%; <10%). Profiles guide monogenic/PRS testing.^13^, ^72^

#### 5.4.1 Focal versus generalized seizures: distinct genetic architectures

Focal (localized onset) versus generalized (bilateral/thalamic) genetics: focal ties rare variants (*KCNT1*, *GRIN2A* ion; *DEPDC5*/*NPRL2/3* mTOR) to structural lesions; generalized common variants in channels/receptors, mirroring focal malformations versus polygenic network bias.^13^

#### 5.4.2 The “second hit” model in focal epilepsies

FCDII invokes germline + somatic “second hit”; ~30% germline‐negative harbor somatic mTOR (*MTOR*/*TSC1*/*2*/*DEPDC5*) variants. Dual hits escalate; low‐frequency (≥1%) suffices for mTOR hyperactivation, cytomegaly, migration flaws, seizures in models.^73^

### 5.5 Syndromic versus isolated epilepsies

Syndromic forms transcend seizures: TSC (*TSC1/2*; 80%–90% seizures + cutaneous/renal/pulmonary)^2^; Dravet (cognition/behavior)^67^; GLUT1 (*SLC2A1*; fasting seizures/dyskinesia/ketogenic‐responsive).^2^, ^67^ Isolated confine to seizures sans systemic: *PRRT2* BFIE recurs infancy, resolves preschool, cognition intact.^62^

### 5.6 Somatic mutation and neuronal genomic hypervariation

#### 5.6.1 Brain somatic mosaic variants in focal epilepsy

Somatic neural variants propel focal epilepsy/FCD; deep sequencing flags mTOR (*MTOR*/*TSC1*/2/*DEPDC5*) in 20%–30% germline‐negative, allele 1%–40%.^74^ Seizures ignite from mutations in ~0.04%–0.09% cells (~8000–9000 neurons), per models.^15^

#### 5.6.2 Neuronal genomic hypervariation and single‐cell mutations

Neurons accrue 1458–1580 unique somatic SNVs, fostering mosaicism^75^; ~16 SNVs/year/neuron, indels in neurodev regulators.^76^ L1 retrotransposons spawn cell‐specific transcripts, steering differentiation.^77^ SGR—RT‐mediated intronless cDNAs—enrich neurodegeneration (e.g., Alzheimer's) sans epilepsy ties; neural RAG1/2 may induce breaks akin to V(D)J, but epilepsy links are unproven (animal behavior models only); human cohorts needed.^78^

### 5.7 Monogenic versus polygenic inheritance: polygenic risk scores

#### 5.7.1 Polygenic risk scores in epilepsy

PRS aggregates common variant effects (OR 1.1–1.3) from GWAS, capturing heritability. Epilepsy PRS predict: top‐decile 2–4‐fold risk versus bottom, variant by type/population; reframes “monogenic” families as polygenic.^49^, ^79^ In this approach, all known genetic variants associated with either increased or decreased susceptibility to the disease are incorporated. For each variant, an individual's genotype, carrying 0, 1, or 2 risk alleles, is recorded. The weighted cumulative effect of these variants on disease susceptibility is then calculated to derive the individual's final PRS.

PRS hold promise for refining individual susceptibility estimates for idiopathic generalized epilepsies, but their predictive power remains constrained by the complex, highly polygenic architecture of these disorders and the limited ancestry diversity of existing datasets. Substantial advances in large‐scale genomic cohorts, cross‐ancestry modeling, and mechanistic interpretation will be required before PRS can achieve clinically actionable accuracy in the near future.^49^

#### 5.7.2 Interaction between polygenic risk and monogenic variants

Polygenic modifies rare variant penetrance/severity; GEFS+ *SCN1B*/*GABRG2* carriers' variance PRS‐explained.^16^ Integrate PRS for monogenic counseling/prognosis.

### 5.8 Genotype–phenotype correlations and phenotype–genotype relationships

#### 5.8.1 EEG signatures and radiogenomics

Genetic epilepsies bear EEG hallmarks: CAE 3‐Hz spike–wave; rolandic centrotemporal spikes.^80^ Radiogenomics links MRI to genes: *TSC1/2* tubers/hamartomas; *DEPDC5/NPRL2/3* FCD; *PCDH19* focal sans MRI.^81^, ^82^, ^83^ Correlations triage testing, now neurology staples.

#### 5.8.2 Metabolic signatures

CSF/serum metabolomics/proteomics fingerprint diseases. TSC: putrescine up, methionine/SAM down (mTOR); serum GFAP up (glial).^84^ GLUT1: CSF glucose/lactate down, gluconic/galactonic/xylose oligos down.^85^ PDE (ALDH7A1): CSF/serum 2‐OAA/6‐oxo‐PIP up.^86^ GAMT: guanidinoacetate up, creatine down.^87^ IESS (*TSC*/*STXBP1*): CSF linoleic down, HLA‐A/*SEZ6L2* down (fatty/neuroimmune).^88^ Biomarkers track activity/response/epileptogenesis, aiding therapies (ketogenic/pyridoxine).

### 5.9 Phenotypic variation with the same gene mutation and genotype–phenotype modifiers

#### 5.9.1 Functional classification of mutations: loss‐of‐function versus gain‐of‐function

LoF (*SCN1A* truncations; Dravet) versus GoF (GEFS+) forecasts gravity.^67^ Residual activity (20%–50% missense milder; <10% severe), locus (voltage‐sensor worse), isoforms modulate; guides blockers.^67^, ^89^

#### 5.9.2 Developmental versus activity‐dependent dysfunction

Developmental (migration/proliferation: *LIS1*/*DCX*/*TUBA1A*/*STXBP1*/*DNM1*/*mTOR*; irreversible dysgenesis, early interventions) versus activity‐dependent (excitability/synaptic: *SCN1A/2A/8A/KCNQ2/3/GABRA1*; mature hyperexcitability, channel/RNA therapies).^90^, ^91^ Age/allele shifts (*SCN2A* GoF infancy developmental; LoF later activity); fuse onset/functional/neuroimaging for precision timing.^90^, ^92^

#### 5.9.3 Mutation‐specific modifiers

Rare modifiers (calcium genes) epistatically/hypostatically interact: *CACNA1A* worsens *SCN1A* via Ca^2+^/excitability (epistasis); *GABRA1* tempers *SCN2A* GoF (hypostasis), family variability.^93^, ^94^

### 5.10 Epigenetics in epilepsy

Epigenetic alterations, including aberrant DNA methylation patterns, chromatin phase transitions, and dysregulation of non‐coding RNAs such as miRNAs, have emerged as critical modulators in the genetic architecture of epilepsy. These multilayered mechanisms influence gene expression without altering the DNA sequence, affecting neuronal excitability, synaptic plasticity, and network synchronization, key determinants of epileptogenesis. Hypermethylation or hypomethylation in genes involved in ion channel regulation, neurotransmitter signaling, and inflammatory pathways can lead to transcriptional repression or activation, thereby predisposing neuronal circuits to hyperexcitability.^95^ Chromatin remodeling and altered euchromatin–heterochromatin dynamics further modulate the accessibility of epileptogenic loci to transcriptional machinery. Concurrently, dysregulated miRNAs orchestrate post‐transcriptional gene silencing, impacting pathways related to neuronal survival and excitatory–inhibitory balance. Technically, these epigenetic modifications are explored through high‐resolution approaches such as whole‐genome bisulfite sequencing (WGBS), reduced representation bisulfite sequencing (RRBS) for methylome profiling, ChIP‐seq and ATAC‐seq for chromatin state mapping, and small RNA‐seq or miRNA microarray analyses for transcriptomic interrogation, collectively offering a comprehensive landscape of epigenomic dysregulation underlying epilepsy.^96^

## 6 DIAGNOSIS OF EPILEPSY WITH GENETIC FOCUS

Epilepsy diagnosis hinges on clinical seizure history, EEG corroboration, and MRI for structural lesions in symptomatic etiologies. Genetic evaluation integrates routinely for early/severe/syndromic cases or phenotype‐driven suspicions, enhancing etiological precision.^12^

### 6.1 Introduction to diagnostic approach: EEG, MRI, and clinical features

Clinical acumen—seizure semiology, frequency, triggers—anchors diagnosis, augmented by EEG for interictal/ictal epileptiform discharges and MRI for epileptogenic foci (e.g., hippocampal sclerosis, malformations). Genetic testing stratifies unexplained or refractory cases, informing prognosis and therapy.^12^

### 6.2 Genetic testing modalities and clinical indications

Tiered modalities span single‐gene Sanger sequencing for phenotype‐gene anchors (e.g., *KCNQ2* exon‐specific in neonatal seizures, *PRRT2* in benign infantile; tandem repeats in *CSTB* for Unverricht‐Lundborg [MIM 254800] or ARX [MIM 308350] syndromes)—now rarely first‐line^97^—to multigene panels (~500 epilepsy loci) for defined phenotypes, balancing depth/cost amid expanding gene rosters.

Whole exome sequencing (WES) scans all coding exons with 15%–45% yields, phenotype/onset‐dependent, ideal for heterogeneous undiagnosed cases.^98^, ^99^, ^100^, ^101^ Whole genome sequencing (WGS) excels in structural/deep intronic/non‐coding variants at premium cost, first‐line for severe neonatal epilepsies or WES failures.^98^, ^99^

### 6.3 Whole exome sequencing strategy: trio, singleton approaches

Trio WES (proband + parents) unmasks de novo variants and inheritance, boosting pathogenicity calls; ~80%–90% severe early DEEs arise de novo (SNV/CNV), rendering trios indispensable.^69^ Singleton suits parental unavailability but risks interpretive ambiguity.

### 6.4 Pitfalls in whole exome sequencing

WES pitfalls encompass shallow coverage missing low‐allele mosaics/GC‐biased regions, alignment artifacts in pseudogenes/homologs, and suboptimal CNV detection—necessitating array‐CGH augmentation.^102^ WGS/long‐read (Nanopore/PacBio) redress via structural/intronic/non‐coding capture, elevating yields in unsolved cohorts.^102^

#### 6.4.1 The current and future use short‐ and long‐read sequencing

Currently, short‐read sequencing (including exome and genome approaches) represents the primary genomic tool in epilepsy diagnostics, offering high accuracy for single‐nucleotide variants and small indels, scalable coverage, and validated clinical pipelines. However, it remains limited in resolving complex structural variants, repetitive regions, and phasing relationships. Long‐read sequencing, though still emerging in clinical practice, overcomes many of these constraints by enabling direct characterization of large structural and repeat expansions, precise haplotyping, and improved detection of mosaic or complex rearrangements. Over time, as costs decline and analytic accuracy improves, long‐read technologies are expected to transition from specialized, second‐tier use to a first‐line or hybrid role alongside short‐read sequencing, achieving comprehensive variant discovery, from single‐base to mega‐base scale, in a single assay.^99^

### 6.5 Challenges in genetic diagnosis

Despite major advances in epilepsy genomics, substantial diagnostic challenges remain. Approximately half of developmental and epileptic encephalopathies and most focal epilepsies remain genetically unresolved following WES or WGS due to undiscovered genes, technical limitations, and incomplete functional interpretation.^103^, ^104^, ^105^ Variants of uncertain significance, non‐coding variation, repeat expansions, and tissue‐restricted somatic mosaicism continue to complicate diagnosis and clinical interpretation. Emerging genomic and functional approaches are expected to improve diagnostic yield and variant classification. Expanded discussion of structural genomic technologies, VUS interpretation strategies, and future diagnostic approaches is provided in Text S2.

### 6.6 Post‐genomic approaches and functional validation

Post‐genomic functional approaches provide critical insight into molecular mechanisms underlying epilepsy and enable validation of candidate variants. Patient‐derived RNA sequencing (RNA‐seq), including analyses from fibroblasts, induced pluripotent stem cells (iPSCs), and brain organoids, can reveal network‐level dysregulation, aberrant splicing events, and variant‐specific transcriptomic effects that contribute to epileptogenesis.^106^, ^107^ Complementing bulk transcriptomics, single‐cell RNA sequencing (scRNA‐seq) enables high‐resolution identification of vulnerable neuronal subtypes. In temporal lobe epilepsy (TLE), this approach has implicated Fezf2^+^ layer 5–6 pyramidal neurons, Cux2^+^ layer 2–3 neurons, and Pvalb^+^ and Sst^+^ interneurons, highlighting disruptions in ion channel regulation, synaptic signaling, and AMPA receptor subunits.^28^ Functional validation is further strengthened using patient‐derived iPSC neurons and cerebral organoid models, which recapitulate disease‐relevant phenotypes and permit drug‐response testing. Notably, *DEPDC5* mosaic organoids reproduce key pathological features—including mTOR pathway hyperactivation, rosette dysmorphia, and neuronal hyperexcitability—that are reversible with rapamycin treatment, closely mirroring focal cortical dysplasia (FCD) pathology.^28^, ^106^

## 7 TRANSLATIONAL APPLICATIONS

### 7.1 Precision medicine and genotype‐guided treatment

Genotype‐directed therapy tailors ASMs (*SCN1A/2A* GoF: Na^+^ blockers; LoF: alternatives; *KCNQ2/3*: K^+^ openers; GABA: enhancers) and predicts responses/adversities. Dravet *SCN1A* avoids exacerbants; TSC mTOR inhibitors (everolimus) mechanistically quell seizures.^105^, ^108^

### 7.2 Gene therapy and advanced molecular therapeutics

Emerging gene and molecular therapies increasingly target the genetic basis of epilepsy through viral delivery, RNA modulation, and regenerative strategies. Non‐integrating lentiviral vectors encoding engineered potassium channels (EKC) are being evaluated for focal neocortical epilepsy in NCT04601974 (Phase I/IIa), with 1–5‐year safety monitoring.^109^ CRISPR activation (CRISPRa), using dCas9‐based transcriptional upregulation, enhances haploinsufficient genes without DNA cleavage; in vivo Kcna1 CRISPRa achieves ~70% seizure reduction with cognitive and transcriptomic rescue.^110^ *ETX101*, an AAV9‐based SCN1A activator targeting GABAergic interneurons, is advancing in pediatric Dravet trials (NCT05419492, NCT06112275, NCT06283212). Antisense oligonucleotides (ASOs) suppress gain‐of‐function variants; KCNT1 gapmer ASOs produce >90% mRNA knockdown with improved seizure control and survival,^111^ while NCT06872125 (EMPEROR) evaluates zorevunersen for major motor seizures in Dravet syndrome.

Regenerative and adjunct approaches are also progressing. NRTX‐1001 allogeneic neural cell therapy for drug‐resistant mesial temporal epilepsy holds RMAT designation, with NCT05135091 (Phase 1/2) and planned NCT06422923 studies.^112^ Dual‐AAV split‐intein systems enable large‐gene replacement (e.g., *SCN1A* with *DLX2.0* targeting), yielding 70%–90% seizure reduction and prolonged protection,^113^ whereas AAV9‐RNAi silencing of DNM1 eliminates lethal seizures and gliosis.^114^ Additional strategies include NPY/Y2 receptor overexpression nearing trials^115^; rAAV‐GDNF or exosome‐based delivery for post‐implant stabilization^116^; miRNA‐targeting therapies such as miR‐134 inhibition and miR‐335‐5p synergy with cannabidiol^117^, ^118^; optogenetic and DREADD neuromodulation approaches^119^; mesenchymal stem cell exosomes to mitigate status epilepticus and neuroinflammation^120^; glucose‐responsive nanoparticles for seizure‐triggered drug release^121^; and TANGO ASOs that enhance NaV1.1 expression and prevent SUDEP in Dravet models, now entering clinical evaluation.^122^

### 7.3 Biomarkers for treatment response and disease progression

Genetic biomarkers forecast responses/adversities (HLA‐B*1502 carbamazepine SJS; CYP2C9 phenytoin dosing).^19^ Serum/CSF/neuroimaging molecular markers gauge activity/epileptogenesis/response.^83^, ^85^ miRNA/proteomic/metabolomic profiles link to frequency/resistance, stratifying therapies.^88^

## 8 KNOWLEDGE GAPS AND FUTURE DIRECTIONS

GWAS capture 39.6%–90% of common variant risk in genetic generalized epilepsy; however, substantial “missing heritability” remains attributable to rare variants, structural alterations, gene–environment interactions, and non‐additive genetic effects.^13^ Resolving this gap will require large‐scale whole‐genome sequencing integrated with deep phenotyping and environmental data. In parallel, the true prevalence of brain somatic mosaicism in refractory focal epilepsy remains undercharacterized, largely due to the technical difficulty of detecting low‐frequency variants in limited brain tissue samples. Application of ultra‐deep, single‐cell, and in situ sequencing to prospectively collected tissues is expected to clarify mosaicism rates and mechanisms.^71^

Another major limitation is incomplete functional annotation, with an estimated 60%–70% of detected variants lacking definitive functional interpretation. Standardized high‐throughput pipelines that integrate in silico prediction, in vitro biophysical assays, and disease models will be essential to accelerate pathogenicity classification.^123^ Despite rapid gene discovery, clinical translation has lagged; for many epilepsy genes, clinical utility remains uncertain and precision therapies still require robust trial validation. Accordingly, randomized studies comparing genotype‐guided management with standard care are critically needed.^124^

Future progress will also depend on integrated multi‐omics strategies combining genomics, transcriptomics, proteomics, metabolomics, and neuroimaging in well‐characterized patient tissues and models to delineate seizure and drug‐resistance networks.^125^ At the therapeutic level, ASM selection remains largely empirical, and pharmacogenomics simulation frameworks that incorporate genetic background, seizure phenotype, and drug–drug interactions could enable truly personalized polytherapy.^18^ In addition, reliable biomarkers predicting pharmacoresistance are still in early development; longitudinal outcome‐linked studies are required to generate clinically actionable signatures.^126^

Artificial intelligence and machine learning models that jointly analyze genetic, clinical, imaging, and EEG data show promise to outperform traditional linear approaches in predicting seizure risk, treatment response, and disease progression, but prospective cohort validation will be essential for clinical deployment.^127^ Important interdisciplinary gaps also persist, including prevention of epileptogenesis after acquired insults such as trauma or infection; management of lifelong comorbidities (psychiatric, sleep, endocrine, and aging‐related outcomes); improved understanding and prevention of SUDEP; reduction of global disparities in low‐ and middle‐income countries; advancement of non‐pharmacologic innovations such as digital seizure detection, neuromodulation, and gene‐ or cell‐based diagnostics; and resolution of ethical, equity, data‐integration, and communication barriers that currently limit implementation of precision epilepsy care.

### 8.1 The global disparity in access to the advanced genetic testing and precision therapies

In developed countries, sequencing tests are relatively inexpensive and widely accessible, whereas in developing countries, such tests may be comparatively costly or even prohibitively expensive. This disparity can reduce the inclination to perform genetic testing and consequently limit the identification of genes and pathogenic variants in these regions. Moreover, the relatively greater influence of environmental factors may further contribute to the lower tendency to utilize genetic testing.

Another global difference concerns the pharmaceutical brands used: the same medication produced by two different companies may vary in its degree of purity, therapeutic efficacy, and side‐effect profile. In addition, delays in diagnosis and in the initiation of treatment may occur. Both of these factors challenge the confirmation of drug‐resistant epilepsy.

## 9 CONCLUSION

The genetic architecture of epilepsy reflects extraordinary complexity, encompassing monogenic forms caused by rare, high‐penetrance variants (such as *SCN1A*, *KCNQ2*, and *CDKL5* mutations in severe early‐onset epilepsies); polygenic forms determined by combinations of common variants with small individual effects; somatic mutations restricted to brain tissue (*MTOR*, *TSC1*, *TSC2* in focal cortical dysplasia); and extensive gene–environment interactions. Understanding seizure and epilepsy genetics requires integrating knowledge across molecular biology, neurophysiology, network dynamics, and population genetics.

Recent advances in genomic sequencing technologies and functional genomics have exponentially expanded identification of epilepsy genes and mechanistic understanding. Yet substantial gaps remain: missing heritability, incomplete variant annotation, undetected somatic mosaicism, and limited clinical translation of genetic discoveries.

Future progress demands large‐scale, well‐phenotyped cohorts with integrated multi‐omics analysis; development of rigorous functional validation methods; and clinical trials demonstrating the value of genotype‐guided treatment. The ultimate goal—personalized, mechanistically informed treatment enabling seizure freedom and preventing developmental complications—requires continued investment in epilepsy genetics research and translation of discoveries into clinical practice. As genetic technologies mature and costs decline, genetic testing will likely become standard in epilepsy evaluation, fundamentally reshaping clinical care toward precision medicine approaches.

## AUTHOR CONTRIBUTIONS

MRS conceived the idea of the manuscript and contributed to writing and figures design. JB contributed to writing the manuscript. MS contributed to writing and prepared the tables. LP was involved in editing and refining the text. PS contributed to writing the manuscript. MS and PS reviewed and verified the accuracy of the content. All authors read and approved the final manuscript.

## CONFLICT OF INTEREST STATEMENT

None of the authors has any conflict of interest to disclose. We confirm that we have read the Journal's position on issues involved in ethical publication and affirm that this report is consistent with those guidelines.

## Supporting information


Table S1.–S3.

Appendix S1.

Text S1.

Text S2.

## Data Availability

Data sharing not applicable to this article as no datasets were generated or analysed during the current study.
