# Supplementary material for: The genetic architecture of epilepsy across molecular mechanisms and clinical heterogeneity
Source: Epilepsia Open. 2026 Aug 19;11(5):1395–412. doi: 10.1002/epi4.70310 (PMC13487861; doi:10.1002/epi4.70310)
Supplement: Supplementary file 1 [file EPI4-11-1395-s001.docx]

Appendix S1:

**Genes involved in monogenic epilepsy based on genes4epilepsy version 2025/09 (**[**https://github.com/bahlolab/Genes4Epilepsy**](https://github.com/bahlolab/Genes4Epilepsy)**).**

1. Ion Channels and Excitability Machinery **(90 genes)**

- Voltage-gated sodium channels (6 genes): SCN10A, **SCN1A**, SCN1B, **SCN2A**, SCN3A, **SCN8A**
- Voltage-gated potassium channels (24 genes): KCNA1, KCNA2, KCNA3, KCNA6, **KCNB1**, KCNB2, KCNC1, KCNC2, KCND1, KCND2, KCND3, KCNH1, KCNH5, KCNH7, KCNJ10, KCNJ11, KCNK4, KCNMA1, KCNN2, **KCNQ2**, **KCNQ3**, **KCNQ5**, **KCNT1**, KCNT2
- Voltage-gated calcium channels and auxiliaries (9 genes): **CACNA1A**, CACNA1B, CACNA1C, CACNA1D, CACNA1E, CACNA1G, CACNA1I, CACNA2D1, **CACNA2D2**
- HCN and other excitability channels (16 genes): **HCN1**, **HCN2**, RYR3, SLC12A5, **SLC13A5**, SLC16A2, SLC1A2, SLC1A4, SLC2A1, SLC45A1, SLC46A1, SLC4A10, SLC6A8, SLC9A6, TRPM3, TRPM6
- Additional channels and transporters (35 genes): ABCC9, ANO4, ATP1A1, ATP13A2, ATP2B1, ATP2B2, ATP6AP2, ATP6V0A2, ATP6V0C, ATP6V1A, ATP6V1B2, ATP7A, CHRNA2, CHRNA4, CHRNA7, CHRNB2, CLCN3, CLCN4, CLCN6, CLDN5, CNNM2, FGF13, KCTD3, KCTD7, NALCN, SLC22A5, SLC25A1, SLC25A12, SLC25A22, SLC35A3, SLC38A3, SLC39A8, SLC5A6, SLC7A6OS, WNK3

2. Inhibitory/Excitatory Neurotransmission and Synaptic Proteins **(89 genes)**

- GABAergic receptors and metabolism (15 genes): ABAT, GABBR2, GABRA1, GABRA2, GABRA3, GABRA4, GABRA5, GABRB1, GABRB2, **GABRB3**, GABRD, **GABRG2**, GAD1, SLC32A1, SLC6A1
- Glutamatergic receptors (10 genes): GRIA1, GRIA2, GRIA3, GRIA4, GRIK2, GRIN1, **GRIN2A**, **GRIN2B**, **GRIN2D**, GRM7
- Synaptic vesicle cycle / release machinery (25 genes): AP1G1, AP2M1, AP3B2, AP3D1, AP4B1, AP4M1, BSN, CPLX1, DNM1, DNM1L, NAPB, NSF, PCLO, RPH3A, SCAMP5, SNAP25, SNAP29, STX1A, **STX1B**, **STXBP1**, **SYN1**, SYNJ1, SYT1, VAMP2, UNC13A
- Cell-adhesion and synaptic organizing proteins (39 genes): ADAM22, ADGRL1, ARHGAP4, ARHGEF9, ASTN1, BLTP1, BORCS5, CAPRIN1, CAPZA2, CASK, CLTC, CNKSR2, CNTNAP1, CNTNAP2, COL18A1, COL3A1, DENND5A, **DLL1**, ESAM, HEPACAM, **IQSEC2**, ITGB4, LGI1, LNPK, LRRC7, NBEA, **NCKAP1**, NRXN1, PCDH12, **PCDH19**, PCDHGC4, **PLXNA1**, PLXNC1, PPFIA3, PPFIBP1, PRRT2, **SEMA6B**, **SHANK3**, STEEP1

3. mTOR/GATOR Pathway and Major Signaling Hubs **(50 genes)**

- mTOR complex and regulators (8 genes): **DEPDC5**, **MTOR**, **NPRL2**, **NPRL3**, STRADA, SZT2, TSC1, TSC2
- PI3K--AKT--RAS--MAPK signaling (17 genes): AKT1, AKT3, BRAF, CSNK2A1, CSNK2B, HRAS, MAP2K1, MAP2K2, MARK2, **MAST1**, **MAST3**, **MAST4**, NRAS, PIK3C2B, PIK3CA, PIK3R2, TAOK1
- Small GTPases and GEFs/GAPs (25 genes): AFF3, AGO1, ARF1, ARF3, ARFGEF1, ARFGEF2, CRADD, **CUL4B**, GNAI1, PTEN, RAB11A, RAB11B, RAB18, RAB39B, RAB3GAP1, RAB3GAP2, RAB5C, RABGAP1, RAC3, RALA, RALGAPA1, RAPGEF2, RHOBTB2, TIAM1, TNK2

4. Transcription Factors and Chromatin/Epigenetic Regulators **(123 genes)**

- Key neurodevelopmental TFs (22 genes): CIC, CREBBP, FOXG1, GLI3, HOXA1, LMX1B, MAF, MYT1L, NEUROD2, OTX2, **PAX6**, PRDM8, PTF1A, RORA, RORB, SHH, SIX3, SOX11, SOX2, SP9, TGIF1, ZEB2
- Chromatin remodelers / writers / erasers (45 genes): ASH1L, BAP1, CHD1, CHD2, CHD3, CHD4, CHD5, DNMT3A, EED, EHMT1, EP400, **GATAD2B**, H3-3A, H3-3B, HDAC3, HDAC8, JARID2, KAT5, KAT6A, KAT8, KDM2B, KDM4B, KDM5A, KDM5C, KDM6A, KMT2A, KMT2B, KMT2C, KMT2D, KMT2E, **KMT5B**, MECP2, MSL2, NSD1, NSDHL, **PHF21A**, **PHF6**, **SETD1A**, **SETD1B**, SMARCA2, SMARCA4, SMARCB1, SMARCC2, SMARCE1, SRCAP
- General transcription / mediator complex (56 genes): ASXL2, **ASXL3**, BAZ2B, BCAS3, BCL11A, **BPTF**, CNOT3, **CNOT9**, CPSF3, DDX53, **DHX16**, **DHX30**, **DHX9**, ERMARD, FAM177A1, GTF3C3, **INTS11**, **INTS8**, JMJD1C, **KANSL1**, **MBD5**, MED11, MED12, MED13L, MED17, MED23, MED27, MEF2C, **NAA10**, **NAA15**, NEXMIF, POGZ, POLR1A, POLR3B, POU3F3, PUF60, SATB1, SATB2, SETD5, **SIK1**, TAF1C, TAF8, **TCF4**, TET3, TFE3, TLK2, TRIP12, **USP9X**, ZBTB18, **ZMIZ1**, ZMYND11, **ZMYND8**, ZNF142, ZNF148, ZNF335, ZNF526

5. Neuronal Migration, Cortical Development and Structural Brain Malformations **(62 genes)**

- Classic migration / lissencephaly genes (36 genes): ASPM, BICD2, CCDC88A, CCDC88C, CENPF, CEP85L, DCX, DYNC1H1, DYNC2H1, KATNB1, KIF1A, KIF2A, KIF5A, KIF5B, KIF5C, KIFBP, MAP1B, **MCPH1**, NDE1, PAFAH1B1, RANBP2, RTTN, RUSC2, TBC1D20, TBC1D24, TBC1D2B, TUBA1A, TUBA8, TUBB, TUBB2A, TUBB2B, TUBB3, TUBB4A, TUBG1, TUBGCP2, TUBGCP6
- Polymicrogyria / cortical dysplasia / axon guidance (26 genes): ADGRG1, **AFDN**, CC2D2A, CCM2, CCND2, CDK5, CDK5RAP2, COL4A1, COL4A2, DCHS1, DMRTA2, EML1, EOMES, FGFR3, GPSM2, KRIT1, LAMC3, MACF1, **PAX6**, PDCD10, **PLXNA1**, **RELN**, **SEMA6B**, TBL1XR1, **WDR62**, ZIC2

6. Synaptic Scaffolding and Postsynaptic Signaling **(36 genes)**

- Scaffolding and postsynaptic proteins (14 genes): CNPY3, CTNNA2, DLG4, EPB41L3, FRRS1L, GPHN, **IQSEC2**, **NCKAP1**, **PHF21A**, **SHANK3**, **SPTAN1**, **SPTBN1**, **SPTBN4**, **SYNGAP1**
- Kinases and phosphatases (22 genes): CAMK2A, CAMK2B, CAMTA1, CDC42BPB, CSNK1E, **MAST1**, **MAST3**, **MAST4**, PAK1, PAK2, PAK3, PDE2A, PIP5K1C, PPP2CA, PPP2R1A, PPP2R5C, PPP2R5D, PPP3CA, RPS6KA3, **SIK1**, YWHAE, YWHAG

7. RNA Processing, Splicing, Translation and tRNA Biology **(88 genes)**

- Spliceosome / snRNA genes (15 genes): CRNKL1, **DHX16**, **DHX30**, **DHX9**, EXOSC3, **INTS11**, **INTS8**, NSRP1, PPIL1, PRPF8, RNU2-2, RNU4-2, RNU4ATAC, SCAF4, TRA2B
- RNA-binding and processing factors (34 genes): ADAR, ADARB1, CELF2, CELF4, DDX39B, DROSHA, **EEF1A2**, EEF1B2, EIF2AK2, EIF2B1, EIF2B2, EIF2B3, EIF2B4, EIF2B5, EIF2S3, EIF3F, EIF4A2, HNRNPH1, HNRNPH2, HNRNPK, HNRNPR, HNRNPU, NOVA2, PTBP1, PUM1, PUS3, RNASEH2A, RNASEH2B, RNASEH2C, RNASET2, SNIP1, SPOUT1, U2AF2, UPF3B
- tRNA synthetases and modification factors (39 genes): AARS1, AARS2, ADAT3, AIMP1, BRAT1, CARS2, CCT3, CCT6A, CCT8, **CNOT9**, CTU2, DOHH, EARS2, EFTUD2, FARS2, IARS2, KARS1, LARS1, NARS1, NARS2, OSGEP, PARS2, QARS1, RARS2, TARS2, TCP1, THG1L, THOC2, TNPO2, TNRC6A, TRIT1, TRMT10A, TSEN15, TSEN2, TSEN34, TSEN54, VARS1, VARS2, WARS2

8. Metabolic Pathways in Epilepsy **(76 genes)**

- Urea cycle / nitrogen metabolism (11 genes): ARG1, ASNS, GLS, GLUD1, GLUL, GOT2, **GPT2**, PAH, PRODH, PTS, QDPR
- Organic acidemias and amino acid metabolism (25 genes): ACOX1, ACY1, BCKDHA, BCKDHB, BCKDK, D2HGDH, DBT, DDC, DHFR, DHPS, FH, GAMT, GATM, GCDH, GLDC, HMGCL, ITPA, L2HGDH, MDH1, MDH2, PCCA, PCCB, PDHX, SUCLA2, SUCLG1
- Vitamin / cofactor metabolism (14 genes): BTD, FOLR1, HLCS, MMACHC, MMADHC, MTHFR, MTHFS, MTR, PANK2, PGK1, PNPO, SLC19A3, TK2, TPK1
- Glycine / serine pathway (26 genes): ALDH3A2, ALDH5A1, ALDH7A1, ALPL, AMPD2, AMT, ASPA, CPT2, DPYD, FCSK, GCH1, GCSH, GLYCTK, GSS, HK1, HPRT1, OGDHL, PHGDH, PLPBP, PSAT1, PSPH, RPIA, SERAC1, SPR, SUOX, UMPS

9. Mitochondrial Respiratory Chain / Energy Metabolism (71 genes)

- OXPHOS complexes and assembly factors (56 genes): AFG3L2, ATP5F1A, ATP5F1E, ATP5MC3, ATP5PO, BCS1L, CLPB, COQ2, COQ4, COQ6, COQ8A, COQ9, COX10, COX15, ECHS1, ETHE1, FASTKD2, FOXRED1, GFM1, HPDL, HSPD1, HTRA2, IBA57, LIAS, LIPT2, MFF, NDUFA1, NDUFA10, NDUFA11, NDUFA13, NDUFA2, NDUFA6, NDUFAF2, NDUFAF3, NDUFAF4, NDUFAF5, NDUFS1, NDUFS2, NDUFS4, NDUFS6, NDUFS7, NDUFS8, NDUFV1, NUBPL, PET100, PMPCB, PNPT1, RMND1, RTN4IP1, SCO1, SCO2, SDHA, SURF1, TIMM50, TSFM, TWNK
- Mitochondrial DNA / mt-encoded (15 genes): AFG2A, AFG2B, BOLA3, CP, GTPBP3, ISCA1, MOCS1, MOCS2, MT-ATP6, MT-ND3, MT-TK, MT-TL1, PDSS2, PTPMT1, TMEM70

10. Lysosomal and Peroxisomal Storage Disorders **(65 genes)**

- Lysosomal enzymes / storage disease genes (37 genes): CLN3, CLN5, CLN6, CLN8, CSTB, CTSD, CTSF, CYP27A1, EPM2A, FUCA1, GALC, GBA1, GLB1, GM2A, GOSR2, GRN, HEXA, HEXB, LYST, MANBA, MFSD8, NAGLU, NEU1, NHLRC1, NHLRC2, NPC1, NPC2, PPT1, PRNP, PSAP, SACS, SERPINI1, SGSH, SUMF1, TPP1, ASAH1, FIG4
- Peroxisomal biogenesis / β-oxidation (28 genes): ABCA2, ACBD6, AGMO, AMACR, ARSA, CERT1, DEGS1, DHCR24, ELOVL4, FA2H, FAR1, HSD17B4, LSS, MBOAT7, PEX1, PEX10, PEX12, PEX13, PEX19, PEX2, PEX26, PEX3, PEX5, PEX6, PEX7, PLA2G6, PLP1, PNPLA8

11. Glycosylation and GPI-Anchor Biosynthesis **(58 genes)**

- N-linked glycosylation / glycan processing (34 genes): ALG1, ALG11, ALG12, ALG13, ALG14, ALG2, ALG3, ALG6, ALG8, ALG9, B3GALNT2, CRPPA, DOLK, DPAGT1, DPM1, DPM2, DPM3, EXT2, FKRP, FKTN, GALNT2, GMPPA, GMPPB, LARGE1, MOGS, MPDU1, PMM2, POMGNT1, POMGNT2, POMK, POMT1, POMT2, RFT1, RXYLT1
- GPI-anchor synthesis (24 genes): B4GAT1, FUT8, GPAA1, NDST1, PGAP2, PGAP3, PIGA, PIGB, PIGC, PIGF, PIGG, PIGH, PIGK, PIGL, PIGM, PIGN, PIGO, PIGP, PIGQ, PIGS, PIGT, PIGU, PIGV, PIGW

12. DNA Repair, Genome Maintenance and Cell Cycle **(21 genes)**

- DNA repair and genome maintenance (10 genes): **CUL3**, LIG3, PNKP, POLG, POLG2, RRM2B, SAMHD1, TDP2, TELO2, TREX1
- Cell cycle and checkpoint control (11 genes): ATRX, CDK13, CDK19, CDKL2, **CDKL5**, FZR1, **MCPH1**, PLK1, RBL2, TBX19, **WDR62**

13. Microtubule/Cytoskeleton/Cell Polarity **(15 genes)**

- Microtubule and cytoskeleton proteins (15 genes): ACTB, ACTG1, ACTL6B, CCDC186, CCDC22, DIAPH1, DNAH14, DYRK1A, FLNA, LMNB2, **SPTAN1**, **SPTBN1**, **SPTBN4**, TBCD, TBCK

14. Cell-Surface Receptors, Adhesion, and BBB/Astroglial Genes **(15 genes)**

- Cell surface and blood-brain barrier (15 genes): **AFDN**, DAG1, **DLL1**, ECM1, GFAP, LAMA2, MLC1, NRROS, OCLN, **RELN**, SCARB2, VLDLR, CSF1R, TREM2, NTRK2

15. Ubiquitination, Proteostasis and Autophagy **(37 genes)**

- Ubiquitination and proteasomal degradation (21 genes): **CUL3**, FBXO11, FBXO28, FBXW7, HECTD1, HECW2, HERC2, KLHL20, MYCBP2, PRMT7, RNF113A, RNF13, **SETD1A**, **SETD1B**, TRAF7, TRIM8, UBE2A, UBE3A, UBR5, UBR7, UBTF
- Autophagy and proteostasis (16 genes): ATAD1, CSTA, DNAJC5, DNAJC6, EPG5, FBXL4, MARCHF6, PLAA, TANGO2, UBA5, UBAP2L, UFC1, UFM1, UFSP2, WDFY3, HTT

16. Core Epileptic Encephalopathy/Synaptopathy Genes **(47 genes)**

- High-impact seizure genes (47 genes): ARX, ATP1A2, ATP1A3, ATP6V0A1, **CACNA1A**, **CACNA2D2**, **CDKL5**, **DEPDC5**, DOCK7, **EEF1A2**, FGF12, **GABRA1**, **GABRB3**, **GABRG2**, GNAO1, GNB1, GNB5, **GPT2**, **GRIN2A**, GRIN2B, **GRIN2D**, **HCN1**, **HCN2**, **KCNA2**, **KCNB1**, **KCNQ2**, **KCNQ3**, **KCNQ5**, **KCNT1**, **MTOR**, **NPRL2**, **NPRL3**, NUS1, **PCDH19**, **SCN1A**, **SCN2A**, **SCN8A**, **SLC13A5**, **SLC2A1**, SLC35A2, ST3GAL3, ST3GAL5, **STX1B**, **STXBP1**, **SYN1**, **SYNGAP1**, VPS13A

17. Remaining Regulatory and Scaffold Hub Genes **(77 genes)**

- Multi-functional regulators and scaffold proteins (77 genes): ADNP, AHDC1, ANK2, ANK3, ANKRD11, ANKRD17, ARID1A, ARID1B, **ASXL3**, AUTS2, **BPTF**, **CUL4B**, CUX2, DEAF1, **DHX30**, EP300, FOXP1, **GATAD2B**, HACE1, HCCS, HCFC1, HIVEP2, IKBKG, IRF2BPL, **KANSL1**, **KMT5B**, LRBA, LRPPRC, **MBD5**, NACC1, **NAA10**, **NAA15**, NAXE, **NBEA**, NF1, NGLY1, NR2F1, NR4A2, OTUD5, OTUD6B, OXR1, PACS1, PACS2, **PHF6**, PURA, RAI1, RFX3, SETBP1, SMC1A, SON, STAG1, STAMBP, **TCF4**, TRIO, **TRIP12**, TRRAP, TSPYL1, **USP9X**, WAC, WASF1, WASHC4, WDR26, WDR37, WDR45, WDR45B, WDR5, WDR73, WWOX, XPR1, YEATS2, ZC4H2, ZDHHC9, **ZMIZ1**, **ZMYND8**, ZNHIT3, ZSWIM6, ATN1

18. Miscellaneous Genes **(104 genes)**

- This category includes genes with emerging or indirect epilepsy associations, or those not clearly aligned with primary mechanisms.
  ADCY5, ADPRS, ADSL, AGA, ALKBH8, ANKH, APC2, ARV1, BMAL1, C12orf57, C19orf12, C2orf69, CAD, CAMSAP1, CASP2, CHKA, COG4, COG6, COG7, COG8, CRELD1, CWF19L1, CYFIP2, DENND2B, DENND5B, DHDDS, DMXL2, EMC1, EMC10, EXOC7, FDFT1, FLVCR1, FRA10AC1, FRMD5, FRYL, GTPBP2, HAX1, HID1, IDH2, IER3IP1, IFIH1, KICS2, KPTN, LMBRD2, MADD, MCM3AP, MINPP1, NCDN, NDP, NECAP1, NEDD4L, NUP214, OCRL, OFD1, OPHN1, P4HTM, PARP6, PCYT2, PDHA1, PHACTR1, PI4K2A, PIDD1, PINK1, PLCB1, PRIMA1, PRUNE1, PTCH1, RERE, ROGDI, SAMD12, SEPSECS, SLC35A1, SMS, SNF8, SNORD118, SNX27, STARD7, SYNE1, TANC2, TBC1D7, TCEAL1, TMEM106B, TMEM184B, TMEM222, TMEM63B, TMX2, TRAK1, TRAPPC10, TRAPPC11, TRAPPC12, TRAPPC4, TRAPPC6B, TTC5, UGDH, UGGT1, UGP2, UNC80, USP18, VPS11, VPS13D, VPS4A, YIF1B, YIPF5, CERS1

Genes which are presented in more than one categories (bolded in the above):

| Gene | Repeat | Gene | Repeat | Gene | Repeat |
| --- | --- | --- | --- | --- | --- |
| DHX30 | 3 | SYN1 | 2 | INTS11 | 2 |
| SCN1A | 2 | DLL1 | 2 | INTS8 | 2 |
| SCN2A | 2 | IQSEC2 | 2 | KANSL1 | 2 |
| SCN8A | 2 | NBEA | 2 | MBD5 | 2 |
| KCNA2 | 2 | NCKAP1 | 2 | NAA10 | 2 |
| KCNB1 | 2 | PCDH19 | 2 | NAA15 | 2 |
| KCNQ2 | 2 | PLXNA1 | 2 | SIK1 | 2 |
| KCNQ3 | 2 | SEMA6B | 2 | TCF4 | 2 |
| KCNQ5 | 2 | SHANK3 | 2 | TRIP12 | 2 |
| KCNT1 | 2 | DEPDC5 | 2 | USP9X | 2 |
| CACNA1A | 2 | MTOR | 2 | ZMIZ1 | 2 |
| CACNA2D2 | 2 | NPRL2 | 2 | ZMYND8 | 2 |
| HCN1 | 2 | NPRL3 | 2 | MCPH1 | 2 |
| HCN2 | 2 | MAST1 | 2 | AFDN | 2 |
| SLC13A5 | 2 | MAST3 | 2 | RELN | 2 |
| SLC2A1 | 2 | MAST4 | 2 | WDR62 | 2 |
| GABRA1 | 2 | CUL4B | 2 | SPTAN1 | 2 |
| GABRB3 | 2 | PAX6 | 2 | SPTBN1 | 2 |
| GABRG2 | 2 | GATAD2B | 2 | SPTBN4 | 2 |
| GRIN2A | 2 | KMT5B | 2 | SYNGAP1 | 2 |
| GRIN2B | 2 | PHF21A | 2 | EEF1A2 | 2 |
| GRIN2D | 2 | PHF6 | 2 | GPT2 | 2 |
| STX1B | 2 | SETD1A | 2 | CUL3 | 2 |
| STXBP1 | 2 | SETD1B | 2 | CDKL5 | 2 |
| CNOT9 | 2 | ASXL3 | 2 | DHX9 | 2 |
| DHX16 | 2 | BPTF | 2 |  | |
