# Supplementary material for: The genetic architecture of epilepsy across molecular mechanisms and clinical heterogeneity
Source: Epilepsia Open. 2026 Aug 19;11(5):1395–412. doi: 10.1002/epi4.70310 (PMC13487861; doi:10.1002/epi4.70310)
Supplement: Supplementary file 2 [file EPI4-11-1395-s002.docx]

6.5_Challenges in Genetic Diagnosis

Despite technological advances, major diagnostic challenges remain. Approximately 50% of developmental and epileptic encephalopathies (DEEs) and 70–80% of focal epilepsies remain genetically unsolved after WES or WGS, attributable to undiscovered genes, technical detection limits, and incomplete functional annotation [1-3]. A large proportion of identified variants, especially missense changes, are classified as variants of uncertain significance (VUS) that require functional validation or long-term clinical correlation, although reclassification rates are rising with advancing technologies [4]. Recurrent copy number variants (e.g., 15q13.3, 16p11.2) confer substantial risk (OR 7–80) as polygenic modifiers; larger structural variants are optimally detected by array-CGH or WGS (~48% diagnostic yield) and often necessitate chromosomal microarray (CMA) after negative exome [5, 6]. Deep intronic (>100 bp from splice junctions) and non-coding regulatory variants that disrupt splicing or cis-regulation evade WES and require WGS combined with RNA-seq or minigene assays [7]. Epigenetic alterations (DNA methylation and histone modifications) modulating gene expression and seizure threshold are undetectable by standard sequencing and necessitate specialized tissue-based analyses [8]. Repeat expansions involving >60 genes cannot be reliably identified by short-read sequencing and demand long-read technologies [9]. Finally, brain-limited somatic mosaicism and gonadal mosaicism frequently escape detection in blood-derived DNA, complicating diagnosis and recurrence risk counseling [10].

The other major microarray platform, SNP array, also enables the detection of uniparental disomy (UPD) in addition to CNVs. Emerging technologies such as optical genome mapping (OGM) represent promising complementary tools. OGM works by applying barcodes to high–molecular-weight DNA molecules and subsequently reading these tagged fragments to identify structural changes. This technique is capable of detecting even balanced chromosomal rearrangements, offering higher resolution than traditional microarrays. Despite their utility, both CMA and OGM have limitations, including lower sensitivity for small intragenic deletions and limited capacity for detecting nucleotide-level changes; this issue is resolve by sequencing methods [11].

VUSs (SNVs/CNVs) remain among the key challenges in medical genetics, where their interpretation demands considerable caution. Although functional studies represent the most definitive approach for elucidating the role of such variants, their implementation is often constrained by technical limitations and resource requirements in many regions worldwide. Consequently, the need for reliable alternative strategies has become evident [12].

Segregation analysis within affected families can yield valuable information about a given variant; however, these results may be misleading when the variant exhibits incomplete penetrance. Refining the initial classification based on ACMG guidelines—such as through manual updates enabled by variant platforms like Franklin or VarSome—can enhance interpretation, particularly when incorporating phenotypic correlations and segregation data [12].

The genomic and structural context of each VUS also warrants careful consideration: for instance, determining whether the variant lies within a specific functional domain, or near a known pathogenic mutation of similar type. Moreover, advances in in silico protein structural analysis and protein–protein docking could, when sufficiently improve in future, serve as useful complementary tools alongside existing pathogenicity prediction methods [13].

Developing a dedicated epilepsy database and submitting VUS findings together with relevant phenotypic details would further contribute to the refinement of variant classification. Nevertheless, utmost caution should be exercised when incorporating VUS data into clinical decision-making, especially in prenatal and preimplantation genetic diagnosis settings.

1.	Aledo-Serrano, A., et al., *Developmental and epileptic encephalopathies after negative or inconclusive genetic testing: what is next?* Journal of Translational Genetics and Genomics, 2021. **5**(4): p. 443-455.

2.	Habela, C.W., K. Schatz, and S.A. Kelley, *Genetic testing in epilepsy: improving outcomes and informing gaps in research.* Epilepsy Currents, 2025. **25**(3): p. 153-157.

3.	Perilli, L., et al., *A tiered decisional framework for genetic epileptic syndromes with or without encephalopathies: from standard care to investigational strategies.* Expert Review of Clinical Pharmacology, 2026. **19**(4): p. 383-401.

4.	Fowler, D.M. and H.L. Rehm, *Will variants of uncertain significance still exist in 2030?* The American Journal of Human Genetics, 2024. **111**(1): p. 5-10.

5.	Chen, W.-L. and H.C. Mefford, *Diagnostic Considerations in the Epilepsies—Testing Strategies, Test Type Advantages, and Limitations.* Neurotherapeutics, 2021. **18**(3): p. 1468-1477.

6.	Montanucci, L., et al., *Genome-wide identification and phenotypic characterization of seizure-associated copy number variations in 741,075 individuals.* Nature communications, 2023. **14**(1): p. 4392.

7.	Robinson, P.N., R.M. Piro, and M. Jager, *Computational exome and genome analysis*. 2017: CRC Press.

8.	Kobow, K., et al., *Epigenetics explained: a topic “primer” for the epilepsy community by the ILAE Genetics/Epigenetics Task Force.* Epileptic Disorders, 2020(2): p. 127-141.

9.	Chen, Z., et al., *Repeat expansion disorders.* Practical Neurology, 2025. **25**(3): p. 204-216.

10.	Rahbari, R., et al., *Timing, rates and spectra of human germline mutation.* Nature genetics, 2016. **48**(2): p. 126-133.

11.	Levy, B., R.D. Burnside, and Y. Akkari, *Optical genome mapping: A new tool for cytogenomic analysis.* Genes, 2025. **16**(8): p. 924.

12.	Richards, S., et al., *Standards and guidelines for the interpretation of sequence variants: a joint consensus recommendation of the American College of Medical Genetics and Genomics and the Association for Molecular Pathology.* Genetics in medicine, 2015. **17**(5): p. 405-423.

13.	Hussein, Y., et al., *Previously defined variants of uncertain significance may play an important role in epilepsy and interactions between certain variants may become pathogenic.* Epilepsia Open, 2024. **9**(6): p. 2443-2453.
