# Supplementary material for: The genetic architecture of epilepsy across molecular mechanisms and clinical heterogeneity
Source: Epilepsia Open. 2026 Aug 19;11(5):1395–412. doi: 10.1002/epi4.70310 (PMC13487861; doi:10.1002/epi4.70310)
Supplement: Supplementary file 3 [file EPI4-11-1395-s003.docx]

**Table 1. Major antiseizure medications categorized by their primary molecular targets, mechanisms of action, and key clinical characteristics**

| Drug Class / Mechanistic Group | Key Agents | Primary Molecular Target(s) | Mechanism of Action | Clinical Role / Key Notes | Ref |
| --- | --- | --- | --- | --- | --- |
| Sodium Channel Blockers | Carbamazepine, Oxcarbazepine, Phenytoin | Voltage-gated Na⁺ channels | Stabilize inactivated channel state, raise firing threshold, suppress high-frequency repetitive firing | Broad efficacy in focal and generalized seizures | [1] |
|  | Phenytoin | Voltage-dependent Na⁺ channels | High-affinity classical blocker | Largely superseded due to adverse effects and nonlinear PK | [1] |
|  | Lacosamide | Na⁺ channels (slow inactivation) | Enhances slow inactivation → reduced neuronal excitability | Improved tolerability vs classical blockers | [1] |
|  | Lamotrigine | Na⁺ channels; presynaptic glutamate release | Dual action: Na⁺ blockade + reduced glutamate release | Broad-spectrum first-line ASM; glucuronidated; induced by phenytoin/carbamazepine → dose adjustment needed | [1] |
| Calcium Channel Blockers | Ethosuximide | T-type Ca²⁺ channels (CACNA1H) in thalamus | Disrupts thalamic rhythmic bursting underlying absence seizures | Highly specific for absence epilepsy | [2] |
|  | Zonisamide | T-type Ca²⁺ channels; Na⁺ channels; carbonic anhydrase | Multi-mechanistic inhibition | Broader spectrum than ethosuximide | [2] |
| Vesicular Protein Modulators | Levetiracetam | SV2A | Reduces readily releasable vesicle pool → decreased neurotransmitter release; activity-dependent vesicle entry | Novel mechanism; no enzyme induction; restores SV2A-related synaptic deficits | [3] |
|  | Brivaracetam | SV2A | Higher-affinity SV2A binding | Greater seizure suppression in some models; potential clinical advantage | [3] |
| Glutamate Receptor Antagonists | Felbamate | AMPA receptors; GABA_A; Na⁺ channels | Multifaceted excitatory suppression and inhibitory modulation | Limited by aplastic anemia and hepatotoxicity | [4] |
|  | Perampanel | AMPA receptors (non-competitive) | Reduces fast excitatory glutamatergic transmission | Effective across multiple seizure types | [4] |
| NMDA Receptor Modulation | Ketamine | NMDA receptor (open-channel block) | Suppresses excitatory transmission | Used in refractory status epilepticus; limited by dissociation | [5] |
| GABA_A Agonists Enhancement and | Benzodiazepines (lorazepam, midazolam, diazepam) | GABA_A receptor | Allosteric potentiation → ↑ Cl⁻ influx | First-line for acute seizures/status; tolerance limits chronic use | [6] |
|  | Barbiturates (phenobarbital, pentobarbital) | GABA_A receptor | Allosteric modulation + direct channel activation | Effective but associated with dependence, cognitive effects, enzyme induction | [6] |
|  | Propofol | GABA_A receptor | Rapid IV agonism | Used in refractory status epilepticus | [7] |
|  | Topiramate | GABA_A; Na⁺ channels; carbonic anhydrase | Multi-mechanism broad action | Broad efficacy but less target specificity | [6] |
| GABA Modulation: Reuptake / Metabolism | Tiagabine | GABA transporter | Inhibits GABA reuptake → ↑ synaptic GABA | Enhances inhibitory tone | [6] |
|  | Vigabatrin | GABA transaminase | Irreversible enzyme inhibition → ↑ brain GABA | Effective in infantile spasms (e.g., CDKL5); risk of irreversible visual field loss | [6] |
|  | Valproate | GABA transaminase; HDAC; Na⁺/Ca²⁺ channels | Multifactorial anticonvulsant effects | Teratogenic; risk of hepatotoxicity and pancreatitis | [6] |
| Potassium Channel Opening | Ezogabine (Retigabine) | Voltage-gated K⁺ channels | Enhances hyperpolarization → raises AP threshold | Limited by retinal pigmentation and urinary toxicity | [8] |
| New-Generation Antiseizure Medications | Cannabidiol (CBD), Fenfluramine | Network excitability pathways | Mechanism-informed therapy effective in developmental and epileptic encephalopathies (Dravet, Lennox–Gastaut) | Supported by RCTs and real-world data; emphasizes exposure–response personalization | [9-11] |
|  | Fenfluramine | Serotonergic pathways (multimodal) | Network-level modulation beyond seizure suppression | Particularly effective in Dravet; reduces overall disease burden | [12] |
|  | Cenobamate | Persistent Na⁺ currents; inhibitory pathways | Dual mechanism targeting network hyperexcitability | High seizure-freedom rates in adult focal DRE | [13] |

**ASM**: Anti-Seizure Medication, **SV2A**: Synaptic Vesicle Protein 2A, **GABA**: Gamma-Aminobutyric Acid, **GABA_A**: Gamma-Aminobutyric Acid Type A Receptor, **NMDA**: N-Methyl-D-Aspartate, **AMPA**: α-Amino-3-hydroxy-5-methyl-4-isoxazolepropionic Acid, **HDAC**: Histone Deacetylase, **CACNA1H**: Calcium Voltage-Gated Channel Subunit Alpha1 H, **CBD**: Cannabidiol, **DRE**: Drug-Resistant Epilepsy, **RCT**: Randomized Controlled Trial

**Table 2. Major Epilepsy-Associated Genes by Functional Category**

| **Functional Category** | **Key Genes** | **Mechanism** | **Associated Phenotypes** | **Clinical Notes** |
| --- | --- | --- | --- | --- |
| **Sodium channels (Na⁺)** | *SCN1A, SCN2A, SCN8A, SCN1B* | Loss/ Gain of function, interneuron vs pyramidal imbalance | Dravet, DEE, GEFS+ | Avoid Na-blockers in SCN1A LoF |
| **Potassium channels (K⁺)** | *KCNQ2, KCNQ3, KCNT1, KCNA1* | Reduced M-current, hyperexcitability | BFNE, DEE, migrating seizures | Retigabine response in KCNQ defects |
| **Glutamate receptors** | *GRIN1, GRIN2A/B/D, GRIA1–4* | Altered Ca²⁺ permeability, excitatory gain | Focal epilepsies, DEE | Consider NMDA modulators |
| **GABA receptors** | *GABRA1, GABRB3, GABRG2* | Reduced inhibition | GEFS+, absence, DEE | Response to GABAergic drugs |
| **mTOR pathway** | *MTOR, DEPDC5, TSC1/2, NPRL2/3* | mTORC1 hyperactivation | FCD, TSC, drug-resistant epilepsy | Everolimus responsive |
| **Synaptic vesicles / SNARE** | *STXBP1, STX1B, SNAP25, DNM1* | Defective vesicle priming/release | Neonatal DEE, spasms | Severe prognosis |
| **Metabolic / mitochondrial** | *POLG, TWNK, SUCLA2, mtDNA* | Energy failure, oxidative stress | Myoclonic epilepsies, encephalopathy | Avoid valproate |
| **Cytoskeleton / cilia** | *AHI1, CEP290, TUBA1A* | Migration defects, ciliary dysfunction | Lissencephaly, FCD | - |

**Table 3. High-Penetrance DEE Genes: Inheritance, Onset, Phenotypes**

| Gene | Variant Type | Inheritance | Typical Onset | Clinical Picture | Notes |
| --- | --- | --- | --- | --- | --- |
| *SCN1A* | LoF missense/nonsense | De novo (80–90%) | 4–12 months | Dravet syndrome | Avoid Na-blockers |
| *KCNQ2* | LoF / GoF | AD or de novo | Neonatal | BFNE ↔ DEE | Retigabine responsive |
| *SCN2A* | GoF early / LoF late | AD, de novo | Days–months | Early DEE | Na-blockers helpful in GoF |
| *STXBP1* | LoF | De novo | Neonatal-infantile | Epileptic encephalopathy | Severe prognosis |
| *CDKL5* | LoF | X-linked dominant | <2 weeks | Spasms, DEE | Early diagnosis crucial |
| *PCDH19* | LoF mosaic | X-linked (female limited) | Infancy | Cluster seizures | Phenotypic variability |
| *DEPDC5* | LoF | AD incomplete penetrance | Childhood-adolescence | Focal epilepsy, FCD | Somatic second-hit common |

1.	Agbo, J., et al., *Therapeutic efficacy of voltage-gated sodium channel inhibitors in epilepsy.* Acta Epileptologica, 2023. **5**(1): p. 16.

2.	Kopecky, B.J., R. Liang, and J. Bao, *T-type calcium channel blockers as neuroprotective agents.* Pflügers Archiv-European Journal of Physiology, 2014. **466**(4): p. 757-765.

3.	Wu, P.-P., et al., *Development of SV2A ligands for epilepsy treatment: a review of levetiracetam, brivaracetam, and padsevonil.* Neuroscience Bulletin, 2024. **40**(5): p. 594-608.

4.	Celli, R. and F. Fornai, *Targeting ionotropic glutamate receptors in the treatment of epilepsy.* Current Neuropharmacology, 2021. **19**(6): p. 747-765.

5.	Sivakumar, S., M. Ghasemi, and S.C. Schachter, *Targeting NMDA receptor complex in management of epilepsy.* Pharmaceuticals, 2022. **15**(10): p. 1297.

6.	Perucca, E., M. Bialer, and H.S. White, *New GABA-targeting therapies for the treatment of seizures and epilepsy: I. Role of GABA as a modulator of seizure activity and recently approved medications acting on the GABA system.* CNS drugs, 2023. **37**(9): p. 755-779.

7.	Prabhakar, H. and M. Kalaivani, *Propofol versus thiopental sodium for the treatment of refractory status epilepticus.* Cochrane Database of Systematic Reviews, 2017(2).

8.	Splinter, M.Y., *Efficacy of retigabine in adjunctive treatment of partial onset seizures in adults.* Journal of Central Nervous System Disease, 2013. **5**: p. JCNSD. S9299.

9.	Cerulli Irelli, E., et al., *Expanding the therapeutic role of highly purified cannabidiol in monogenic epilepsies: A multicenter real‐world study.* Epilepsia, 2025.

10.	Arzimanoglou, A., et al., *Epilepsy and cannabidiol: a guide to treatment.* Epileptic Disorders, 2020(1): p. 1-14.

11.	Lattanzi, S., et al., *Third-generation antiseizure medications for adjunctive treatment of focal-onset seizures in adults: a systematic review and network meta-analysis.* Drugs, 2022. **82**(2): p. 199-218.

12.	Lattanzi, S., et al., *Adjunctive cenobamate for focal-onset seizures in adults: a systematic review and meta-analysis.* CNS drugs, 2020. **34**(11): p. 1105-1120.

13.	Dini, G., et al., *Reintroducing fenfluramine as a treatment for seizures: current knowledge, recommendations and gaps in understanding.* Neuropsychiatric Disease and Treatment, 2023: p. 2013-2025.
