# Supplementary material for: The genetic architecture of epilepsy across molecular mechanisms and clinical heterogeneity
Source: Epilepsia Open. 2026 Aug 19;11(5):1395–412. doi: 10.1002/epi4.70310 (PMC13487861; doi:10.1002/epi4.70310)
Supplement: Supplementary file 4 [file EPI4-11-1395-s004.docx]

4.Treatment Refractoriness and Anti-Seizure Drug Mechanisms

To provide structured context for the major antiseizure medication (ASM) classes and their mechanisms of action, the most clinically relevant agents are summarized in Supplementary Table-2. Despite substantial advances in ASM development over the past two decades, approximately 30% of individuals with epilepsy remain pharmacoresistant after adequate trials of two or more appropriately selected ASMs, underscoring the importance of mechanism-informed therapy selection [1]. A schematic overview of the principal molecular targets and pathways modulated by these drugs is illustrated in Figure 2. Continued integration of molecular pharmacology with genetic and clinical stratification is expected to further refine precision treatment approaches in epilepsy.

4.2_Pharmacokinetic Considerations in Anti-Seizure Drug Response

The pharmacokinetic handling of ASMs—including metabolism, genetic variability, and efflux transport—critically governs systemic drug levels, drug–drug interactions, and the emergence of pharmacoresistance.

4.2.1_Hepatic Metabolism and Enzyme Induction

First-line ASMs (phenytoin, carbamazepine, valproate) potently induce CYPs (3A4, 2C9, 2C19), hastening co-drug/hormone/endogenous clearance and spawning interactions that erode efficacy [2, 3].

Autoinduction is well recognized with phenytoin and carbamazepine, where initially slow accumulation is followed by enhanced metabolic clearance, necessitating dose adjustment. Newer levetiracetam/perampanel/lacosamide sidestep induction, streamlining polypharmacy [2, 3].

4.2.2_Genetic Polymorphisms in Drug Metabolism

Interindividual ASM flux stems from CYP polymorphisms; *CYP2C9*/*2C19* are pivotal for phenytoin/carbamazepine. CYP2C9*3 (frequent in Europeans, scarce in Asians) encodes low-activity enzyme, priming toxicity via elevated levels [3]. Loss-of-function carriers accrue higher concentrations at norms [3]. Meta-analyses show poor/intermediate metabolizers need 2.1–3.4 mg/kg/day phenytoin for targets, versus extensive [4].

4.2.3_Blood-Brain Barrier Transporters and CNS Penetration

ATP-binding cassette (ABC) transporters at the blood–brain barrier (including ABCB1/P-gp, MRPs, and BCRP) actively efflux many ASMs; epilepsy overexpression (e.g., 4–6x P-gp/MRP-1 in temporal lobe) fosters resistance by slashing brain entry [5, 6]. *ABCB1* polymorphisms modulate expression/activity, prognostic for responses [7].

4.3_Pharmacodynamic Considerations and Drug Adverse Effects

Pharmacodynamic resistance may emerge from target adaptations, mechanism–drug mismatches, and off-target effects, often compounded by network and metabolic alterations.

4.3.1_Target Modification and Adaptive Responses

Chronic ASMs provoke network adaptations eroding efficacy, e.g., benzodiazepine tolerance from GABA_A downregulation/subunit reconfiguration [8]. Target-level variants further sabotage binding [9].

4.3.2_HLA-Associated Adverse Drug Reactions

HLA-B*1502 (up to 50% in East/Southeast Asians, rare elsewhere) spikes SJS/TEN risk ~115-fold with aromatic ASMs (carbamazepine/phenytoin/oxcarbazepine/lamotrigine) via T-cell peptide mishandling [10]. At-risk screening preempts most reactions [10].

4.3.3_Neural Network Plasticity and Long-Term Drug Effects

Sustained ASMs reshape synapses, spines, connectivity—conferring benefits but cognitive/behavioral detriments, especially from older non-selectives [11].

4.3.4_Metabolic and Mitochondrial Effects of ASMs

Valproate/topiramate compromise mitochondrial bioenergetics at supratherapeutic doses, driving weight/metabolic woes/hepatotoxicity/pancreatitis; mitochondrial/metabolic disorder hosts bear heightened susceptibility [12].

1.	Chen, Z., et al., *Epidemiology of epilepsy and seizures*. 2023, Frontiers Media SA. p. 1273163.

2.	Zaccara, G. and V. Franco, *Pharmacokinetic interactions between antiseizure and psychiatric medications.* Current neuropharmacology, 2023. **21**(8): p. 1666-1690.

3.	De Bellis, M., et al., *Adverse effects of antiseizure drugsmedications: a review of the impact of pharmacogenetics and drugs interactions in clinical practice.* Frontiers in Pharmacology, 2025. **16**: p. 1584566.

4.	Kanjanasilp, J., et al., *A meta-analysis of effects of CYP2C9 and CYP2C19 polymorphisms on phenytoin pharmacokinetic parameters.* Pharmacogenomics, 2021. **22**(10): p. 629-640.

5.	Urquhart, B.L. and R.B. Kim, *Blood− brain barrier transporters and response to CNS-active drugs.* European journal of clinical pharmacology, 2009. **65**(11): p. 1063-1070.

6.	Ma, A., et al., *P-glycoprotein alters blood–brain barrier penetration of antiepileptic drugs in rats with medically intractable epilepsy.* Drug Design, Development and Therapy, 2013: p. 1447-1454.

7.	Hung, C.-C., et al., *Functional evaluation of polymorphisms in the human ABCB1 gene and the impact on clinical responses of antiepileptic drugs.* Pharmacogenetics and genomics, 2008. **18**(5): p. 390-402.

8.	Vinkers, C.H. and B. Olivier, *Mechanisms underlying tolerance after long‐term benzodiazepine use: a future for subtype‐selective GABAA receptor modulators?* Advances in Pharmacological and Pharmaceutical Sciences, 2012. **2012**(1): p. 416864.

9.	Cárdenas-Rodríguez, N., et al., *Genetic variations associated with pharmacoresistant epilepsy.* Molecular medicine reports, 2020. **21**(4): p. 1685-1701.

10.	Tham, K.M., J.J.L. Yek, and C.W.Y. Liu, *Unraveling the genetic link: an umbrella review on HLA-B* 15: 02 and antiepileptic drug-induced Stevens–Johnson syndrome/toxic epidermal necrolysis.* Pharmacogenetics and Genomics, 2024. **34**(5): p. 154-165.

11.	de Souza, D.L.S., et al., *Brain neuroplasticity after treatment with antiseizure: A review.* Clinical Psychopharmacology and Neuroscience, 2023. **21**(4): p. 665.

12.	Finsterer, J. and S. Zarrouk Mahjoub, *Mitochondrial toxicity of antiepileptic drugs and their tolerability in mitochondrial disorders.* Expert opinion on drug metabolism & toxicology, 2012. **8**(1): p. 71-79.
